# Presenting Psychiatric and Neurological Symptoms and Signs of Brain Tumors before Diagnosis: A Systematic Review

**DOI:** 10.3390/brainsci11030301

**Published:** 2021-02-27

**Authors:** Fatima Ghandour, Alessio Squassina, Racha Karaky, Mona Diab-Assaf, Paola Fadda, Claudia Pisanu

**Affiliations:** 1 Department of Biomedical Sciences, Division of Neuroscience and Clinical Pharmacology, University of Cagliari, 09042 Monserrato, Italy; f.ghandour@studenti.unica.it (F.G.); squassina@unica.it (A.S.); claudia.pisanu@unica.it (C.P.); 2 EDST, Pharmacology and Cancerology Laboratory, Faculty of Sciences, Lebanese University, Beirut 1500, Lebanon; mdiabassaf@ul.edu.lb; 3 Drug-Related Sciences Department, Faculty of Pharmacy, Lebanese University, Hadath 1500, Lebanon; racha.karaky@ul.edu.lb; 4 Centre of Excellence “Neurobiology of Addiction”, University of Cagliari, 09042 Monserrato, Italy; 5 CNR Institute of Neuroscience-Cagliari, National Research Council, 09042 Monserrato, Italy; 6 National Institute of Neuroscience (INN), 10126 Turin, Italy

**Keywords:** brain tumor, intracranial tumor, neuropsychiatry, behavior, early diagnosis

## Abstract

Brain tumors can present with various psychiatric symptoms, with or without neurological symptoms, an aspect that complicates the clinical picture. However, no systematic description of symptoms that should prompt a neurological investigation has been provided. This review aims to summarize available case reports describing patients with brain tumors showing psychiatric symptoms before brain tumor diagnosis, in order to provide a comprehensive description of these symptoms as well as their potential relationship with delay in the diagnosis. A systematic literature review on case reports of brain tumors and psychiatric symptoms from 1970 to 2020 was conducted on PubMed, Ovid, Psych Info, and MEDLINE. Exclusion criteria comprised tumors not included in the World Health Organization (WHO) Classification 4^th^ edition and cases in which psychiatric symptoms were absent or followed the diagnosis. A total of 165 case reports were analyzed. In a subset of patients with brain tumors, psychiatric symptoms can be the only manifestation or precede focal neurological signs by months or even years. The appearance of focal or generalized neurological symptoms after, rather than along with, psychiatric symptoms was associated with a significant delay in the diagnosis in adults. A timely assessment of psychiatric symptoms might help to improve early diagnosis of brain tumors.

## 1. Introduction

Indeed, any type of tumor is considered as an insidious hazard of life, but the uncontrolled proliferation and abnormal growth of cells inside the brain—the organ that makes us who we are—creates an extraordinarily potent threat to our being. Brain tumors are rare compared with other life-threatening diseases. However, ”rare” is a relative term; as 321,000 persons are diagnosed each year with brain and nervous system cancer globally [1]. More than two thirds of them will die and the others might show some restrictions in function [1].

Despite the fact that brain tumors account for a small proportion of all tumors (1.6%) and tumor-related deaths (2.5%) [2], most of these tumors are fatal if they do not receive a timely diagnosis. Even benign tumors can be lethal or at least interfere with brain functions that are essential for daily living. Prognosis is related to their location, ability to infiltrate locally, and propensity to malignant transformation [3]. Brain tumors not only occur in adults but also among children. In fact, brain tumors are the most frequent solid tumors in children and the second most frequent type of cancer, after leukemia [4]. Malignant and benign tumors’ diagnosis poses difficulties, and this is reflected in their symptom interval, defined as the period of time elapsed between the onset of symptoms and the diagnosis. Indeed, studies report a mean symptom interval of at least 14 weeks [5,6,7,8,9].

Several factors contribute to make early diagnosis of brain tumors challenging. Most of the initial symptoms are non-specific and resemble other more common and non-threatening illnesses. Before being diagnosed, patients may have headache, nausea, lethargy, or they may lose weight. Especially in the case of psychiatric symptoms, as they do not represent pathognomonic features of brain tumors, the underlying organic cause may be overlooked, making the diagnosis in the early course often extremely difficult [10,11]. 

Various psychiatric symptoms have been attributed to brain tumors since the 1930s [12]. Keschner et al. reported that, among 530 patients with brain tumors, 78% had psychiatric symptoms, while 18% presented these symptoms exclusively as the first clinical manifestation of the tumor. At that time, and for the next several decades, brain tumors were only recognized by neurological symptoms that vary depending on multifactorial causes. Furthermore, there were no computerized tomography (CT) scans nor efficient multimodality treatment of brain tumors until the 1970s [13,14]. Before the availability of imaging techniques, the psychiatric symptoms of the brain tumors were less well characterized. They had been typically thought to occur along with focal neurological symptoms, and patients were often considered to have a neuropsychiatric disorder. However, focal neurological and psychiatric symptoms do not always coincide. The misrecognition of this concept leads to significant delays in diagnosis and treatment, which may result consequently in irreversible neurological damage or even death. Individuals with a family history of two or more close relatives with the disease, carriers of certain genetic mutations (including the cell checkpoint genes TP53 and CHEK2 (Li-Fraumeni syndrome), neurofibromatosis (NF1, NF2), tuberous sclerosis (TSC1, TSC2), and the DNA repair genes (Turcot’s syndrome) [15]), as well as people with weakened immune systems [1], show a higher risk of developing brain tumors. However, some people who develop brain tumors do not fall into these categories. Non-genetic risk factors for developing sporadic brain tumors include exposure to high ionizing radiation, lack of exercise, head trauma, seizures, administration of centrally acting drugs like hypnotics and anti-histaminergic drugs, as well as exposure to harmful industrial chemicals [1,3].

Although brain tumors are commonly associated with focal neurological symptoms, these may represent “the sound of the collision with the iceberg”. It was also suggested that, in addition to these typical symptoms, the clinical presentation may also include “the tip of the iceberg”, consisting of some psychiatric manifestations that, in most cases, precede the neurological ones. Many studies have focused on the psychiatric condition after brain tumor diagnosis, where substantial neuronal damage was already established. In this systematic literature review, we searched for case reports reporting the onset of psychiatric symptoms before or along with brain tumor diagnosis, in an attempt to shed the light over all possible relevant, and often neglected, psychiatric and neurological symptoms that may occur in brain tumor patients, and to identify features associated with delay in the diagnosis of brain tumors.

## 2. Materials and Methods

We conducted a systematic review in accordance with the PRISMA guidelines [16]. The literature search was conducted with the aim to find published reports of brain tumors and psychiatric symptoms from 1970 up to December 31, 2020. The search was conducted on PubMed, Ovid, Psych Info, and MEDLINE, using the key words (“brain cancer” OR “brain tumor” OR “intracranial tumor”) AND (“psychiatry” OR “psychiatric” OR “depression” OR “apathy” OR “anxiety” OR “mania” OR “psychosis” OR “schizophrenia” OR “personality” OR “behavior”). Bibliographic references of relevant articles were reviewed for additional studies. 

### 2.1. Eligibility Criteria and Study Selection

Case reports and case letters in any population, involving any age group and describing cases in which a patient with a diagnosis of brain tumor had showed psychiatric symptoms or disorders before or along the diagnosis of the brain tumor were considered eligible for the present review. Types of brain tumors considered eligible included those coded in the World Health Organization (WHO) Classification of Tumors of the central nervous system (CNS), 4th edition [17]. Exclusion criteria comprised reviews, opinions, and studies in which psychiatric symptoms were absent or appeared after the diagnosis of the brain tumor. Studies published in languages other than English were included if the full text was available to be translated completely.

Upon completion of the electronic database search, titles and abstracts of the identified articles were read and assessed for their suitability to be included in the review. Subsequently, the full-text of the articles deemed suitable was retrieved for further examination. Psychiatric symptoms were coded according to the classification system of Madhusoodanan et al. [18], which includes seven main categories (depressive symptoms, apathy, manic symptoms, psychosis, personality changes, eating disorders, and a miscellaneous category) to which we added an 8th category: anxiety. It is worthy to note that apathy is distinguished from depression. “Apathy” manifests with absence or lack of motivation, feeling, emotion, interest, concern, or goal-directed behavior motivation not attributable to a decreased level of consciousness, cognitive impairment, or emotional distress, but sadness, anhedonia are not present as in the “depression” state [19]. Personality changes may include disinhibition, hypersexuality, and aggressive behaviors, while the miscellaneous category comprise the ambiguous, less common and atypical psychiatric symptoms such as pathological laughter [20], and new-onset pedophilia [21]. Some reports may be included in more than one category due to combination of symptoms.

In addition to the psychiatric symptoms, for each case we collected the following information: age at the tumor diagnosis, gender, tumor type (according to WHO Classification of Tumors of the CNS, 4th edition, [17]) and location (categorized into supratentorial and infratentorial location, based on the PDQ Pediatric Treatment Editorial Board [22]), generalized and focal neurological symptoms, the chronological onset of neurological symptoms (along with or after the onset of psychiatric symptoms), months between the onset of psychiatric symptoms and tumor diagnosis, and the status of the psychiatric symptoms after tumor resection or treatment (resolved/improved or remained). 

### 2.2. Analysis of Features of Included Studies

All analyses were stratified according to age at diagnosis (pediatric cases: age < 18 years; adult cases: age between 18 and 64 years; and older adult cases: age ≥ 65 years). Differences in the frequency of psychiatric and neurological symptoms between these age groups were analyzed using Fisher’s Exact test. In each age group, the association between quantitative (age at diagnosis, delay between the onset of psychiatric symptoms and tumor diagnosis) or categorical variables (gender, tumor type, tumor location, type of symptoms, resolution of psychiatric symptoms after tumor treatment) and the chronological onset of neurological symptoms (along with or after the onset of psychiatric symptoms) was assessed using Mann-Whitney U test or Fisher’s Exact test, respectively. As only 2 out of 165 cases showed both supratentorial and infratentorial location, these cases were excluded from analyses conducted on tumor location. We also evaluated the association between specific symptoms and the delay between the onset of psychiatric symptoms and tumor diagnosis using Mann-Whitney U test. In the adult group, we also constructed a linear regression model with delay in diagnosis as the outcome, and age, gender, tumor type, tumor location and specific symptoms as predictors. This analysis was not conducted in the pediatric and older adult group due to the limited number of included cases. A *p*-value < 0.05 was considered to be significant. Analyses were conducted using SPSS version 24 (IBM Corporation, Armonk, NY, USA). 

## 3. Results

**Figure 1:**
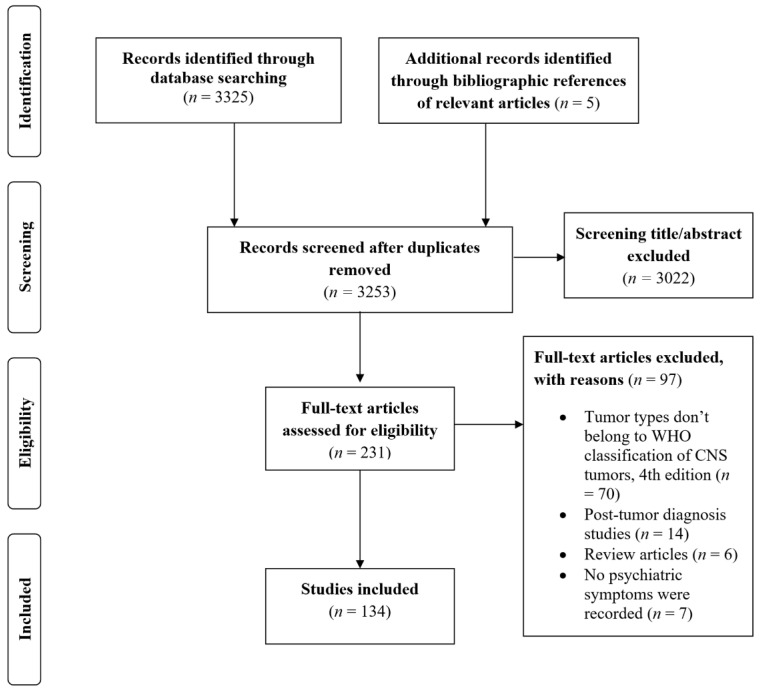
Flow chart showing the selection process of studies.

The search retrieved a total of 3325 studies, while five additional studies were identified through bibliographic references from relevant articles (Figure 1). After exclusion of 77 duplicates, 3253 articles were evaluated. After screening the titles and abstracts, 3022 studies were excluded, and the full-text of 231 articles was further assessed for eligibility. At this stage, 97 articles were excluded being cases of tumors not included in the WHO Classification of Tumors of the CNS 4^th^ edition [17], post-tumor diagnosis studies, review articles, or cases with no psychiatric symptoms. A total of 134 studies were included. Some articles reported more than one case and this led to a total number of cases of 165.

### 3.1. Cases Characteristics

Table 1 and Supplementary Tables S1–S3 show the characteristics of the 165 cases in which psychiatric symptoms appeared before diagnosis of the brain tumor in pediatric cases (33 cases, age at diagnosis <18 years), adults (108 cases, 18 to 64 years) and older adults (24 cases, ≥65 years). The age of the patients ranged between 3 and 86 years, and 88 (53.3%) were women. The average age at which the brain tumors were diagnosed was 40.7 years (standard deviation, S.D. = 20.6). The average time between the onset of psychiatric symptoms and the diagnosis of the brain tumors was 2.6 years (S.D. = 4.4 years), with a range from 1 week [23] to 27 years [24]. Only 13 (7.9%) of the 165 cases were diagnosed within 30 days after the onset of psychiatric symptoms.

Frequencies of specific psychiatric or neurological symptoms are shown in Supplementary Table S4. Eating disorders and apathy showed significant differences in frequencies among age groups, with the former being more frequent in pediatric cases (χ^2^ = 32.59, *p* < 0.0001, with 16, 11 and 0 cases in the pediatric, adult and older adult cases, respectively) and the latter in older adults (χ^2^ = 9.95, *p* = 0.007, with one, 16 and eight cases in the pediatric, adult and older adult cases, Supplementary Table S4). Among neurological symptoms, cognitive deficits were more frequent among older adults (χ^2^ = 10.74, *p* = 0.005, with eight, 41 and 16 cases in pediatric, adult and older adult cases), while nausea and vomiting among pediatric cases (χ^2^ = 17.07, *p* = 0.0002, with 16, 19 and two cases in the three age groups). No symptom was significantly associated with gender. 

Next, we assessed differences in frequencies of psychiatric or neurological symptoms based on tumor location (Supplementary Table S5). In pediatric cases, nine children presented with headaches and ocular impairments in a sample including 21 supratentorial tumors compared to none in a sample including nine cases of infratentorial tumors (43% vs. 0%, χ^2^ = 5.51, *p* = 0.029 for both). In adults, sleep disturbances were more frequent in patients with supratentorial tumors, being reported in 31 out of 91 supratentorial tumors cases compared to one out of 17 infratentorial cases (34% vs. 6%, χ^2^ = 5.46, *p* = 0.02). We also observed a trend for lower frequency of miscellaneous psychiatric symptoms among cases with supratentorial (six cases out of 91) compared to infratentorial tumors (four cases out of 17, 7% vs. 24%, χ^2^ = 4.80, *p* = 0.049). While in older adults, nausea or vomiting were less frequent in cases with supratentorial compared to infratentorial tumors (0 vs. two cases, χ^2^ = 13.93, *p* = 0.013), these results are not reliable do to the extremely small number of participants included in this group. No other neurological or psychiatric symptoms were associated with tumor location (Supplementary Table S5).

In most cases, patients were started on psychotropic treatments after the appearance of psychiatric symptoms. However, patients usually did not respond to treatments despite repeated adjustments. The diagnosis of the tumor was suspected in most cases only after neurological problems were noted, or when the psychiatric symptoms got worse. On the other hand, the psychiatric symptoms were resolved or improved in 93 out of 100 cases after the surgical removal of the tumor or its management through chemotherapy and radiotherapy (Supplementary Table S6). Taken together, these data underline the need to consider brain tumors in differential diagnosis in patients showing psychiatric symptoms. 

### 3.2. Onset of Neurological Symptoms Compared to Psychiatric Symptoms in Pediatric Cases

Only two out of 33 cases among the pediatric group exclusively showed psychiatric symptoms, while eight cases only showed both psychiatric and generalized neurological symptoms (Supplementary Table S6). The average delay between the onset of psychiatric symptoms and tumor diagnosis was 17.9 months (S.D. = 20.6 months). After the resection or treatment of the tumor, the psychiatric symptoms remitted or improved in 23 cases and persisted in two cases. Among the cases reporting data on the chronological onset of neurological compared to psychiatric symptoms, 18 reported the incidence of generalized neurological symptoms along with psychiatric symptoms, while nine after the onset of psychiatric symptoms. Cases in which generalized neurological symptoms appeared along with psychiatric symptoms were younger (Mann-Whitney’s U = 42.5, *p* = 0.046), while there was no significant difference based on gender, tumor type or location, time from onset of psychiatric symptoms to diagnosis, resolution of symptoms after treatment or frequency of specific symptoms (Table 2). As regard to focal neurological symptoms, in four cases they appeared along with and in 19 after psychiatric symptoms (Table 3). There was no significant difference between these groups as regard to gender, age at diagnosis, tumor type or location (Table 3). We observed a non-significant trend for longer delay in the diagnosis when neurological symptoms appeared after psychiatric symptoms (Mann-Whitney’s U = 15.00, *p* = 0.07). Patients in which generalized neurological symptoms appeared along with psychiatric symptoms showed higher frequency of sleep disturbances (χ^2^ = 6.01, *p* = 0.04) and seizures (χ^2^ = 10.41, *p* = 0.024) (Table 3). However, these results are limited by the extremely limited number of patients included in either group.

### 3.3. Onset of Neurological Symptoms Compared to Psychiatric Symptoms in Adult Cases

Among the cases diagnosed in adults, 15 showed exclusively psychiatric symptoms, while 43 psychiatric and generalized neurological symptoms without focal neurological symptoms (Supplementary Table S6). The average delay between the onset of psychiatric symptoms and the diagnosis of brain tumor was 37 months (S.D. = 59.3 months). In 62 cases the psychiatric symptoms remitted or improved after treatment of the underlying brain tumor, while in five cases the symptoms persisted. Among the cases reporting data on the chronological onset of neurological compared to psychiatric symptoms, 39 presented generalized neurological symptoms along with psychiatric symptoms and 40 after the onset of psychiatric symptoms. While there was no significant difference in age and gender among the two groups, cases in which neurological symptoms appeared after psychiatric symptoms showed a significantly higher delay in the diagnosis (median: 24 compared to 5 months; Mann-Whitney’s U = 381.5, *p* = 0.002, Table 2). Additionally, we observed marginally significant differences in tumor type frequencies, with “meningiomas” and “diffuse astrocytic and oligodendroglial tumors” being the most frequent types in patients in which neurological symptoms appeared along with or after psychiatric symptoms, respectively (Table 2). Cognitive deficits (χ^2^ = 6.22, *p* = 0.023, 26 vs. 15 cases) and sleep disturbances (χ^2^ = 5.30, *p* = 0.038, 21 vs. 11 cases) were more frequent in patients in which neurological symptoms appeared along with psychiatric symptoms (Table 2), while no differences were observed for other symptoms.

As regard to focal neurological symptoms, 16 cases showed focal neurological symptoms along with psychiatric symptoms and 49 after the psychiatric symptoms’ onset. As in the case of generalized neurological symptoms, higher delay in the diagnosis was observed in patients in which neurological symptoms appeared after psychiatric symptoms (median: 18 compared to 3 months; Mann-Whitney’s U = 161.5, *p* = 0.012, Table 3). Patients in which neurological symptoms appeared along with psychiatric symptoms showed higher frequency of motor deficits (χ^2^ = 7.37, *p* = 0.008, 23 vs. 13 cases), while no other significant difference was observed between the two groups. 

### 3.4. Onset of Neurological Symptoms Compared to Psychiatric Symptoms in Older Adult Cases

Among cases diagnosed in the older adult group, 10 presented just psychiatric and generalized neurological symptoms (Supplementary Table S6). The average duration of time elapsed between the onset of psychiatric symptoms and diagnosis of the tumor was 23.2 months (S.D. = 54.8 months). The eight cases that recorded the status of the psychiatric symptoms after tumor resection or treatment were all resolved or improved.

Among the 23 cases who presented generalized neurological symptoms, in 18 cases they appeared together with psychiatric symptoms and in 6 after the onset of psychiatric symptoms. There was no significant differences in age, gender, tumor type or location as well as delay in the diagnosis between the two groups. Ocular impairments and seizures (χ^2^ = 7.49, *p* = 0.043 for both, zero vs. two patients) were more frequent in cases in which neurological symptoms appeared after psychiatric symptoms, although these analyses are not reliable due to the very low number of participants showing these symptoms (Table 2). 

As regard to focal neurological symptoms, six cases presented them along with psychiatric symptoms, and eight after the appearance of psychiatric symptoms. The two groups did not show significant differences in any of the tested characteristics.

### 3.5. Symptoms Associated with Delay in Diagnosis

In pediatric cases, personality changes and eating disorders were associated with decreased and increased delay in diagnosis in unadjusted analyses (Table 4) while analyses adjusted for potentially confounding factors were not conducted due to the limited number of cases. In adults, we observed cognitive deficits to be negatively associated with the delay in diagnosis both using unadjusted analyses (Mann-Whitney U = 619.5, *p* < 0.0001) as well as a linear regression model adjusted for age, gender, type of tumor and tumor location (beta = −0.38, *p* = 0.0004). Eating disorders were associated with increased delay in diagnosis in unadjusted analyses (Mann-Whitney U = 246.5, *p* = 0.007) as well as in the adjusted linear regression model (beta = 0.30, *p* = 0.02). No other symptom was associated with delay in diagnosis (Table 4). Finally, in older adults, ocular impairments and seizures were associated with increased delay in diagnosis in unadjusted analyses (Table 4). As for pediatric cases, analyses adjusted for potentially confounding factors were not conducted due to the limited number of included cases.

## 4. Discussion

Organic disorders that produce acute psychiatric manifestations or mimic specific psychiatric disorders have been reported to account for up to 10% of all psychiatric disorders [25]. However, it is often difficult to differentiate between a primary psychiatric disorder and one that is secondary to an organic disease. The association between brain tumors or other organic brain lesions and a wide range of psychiatric manifestations (e.g., depression, apathy, anxiety, psychosis, mania, personality changes or eating disorders) has been revealed more than 80 years ago [12]. In the cases included in our review, most patients were diagnosed with psychiatric diseases and treated accordingly, with a complete unawareness of the underlying disease or a significant delay in diagnosis. Early diagnosis of intracranial tumors is clearly desirable, but sometimes patients may have minimal or no neurological symptoms and signs. In such cases, psychiatric symptoms may be the first and only clue. However, the diagnosis of the underlying organic brain disorder is complicated by the relative rare incidence of brain tumors, the fact that the brain is relatively silent as regard to pain [26] and that certain types of slowly growing tumors are commonly found at sites likely to cause mental changes early, such as meningiomas involving the frontal lobes [27,28] or gliomas involving the anterior midline structures [29], while neurological signs may appear later.

In typical cases, patients with brain tumors develop focal (e.g., motor deficits, seizures, ocular impairments, urinary incontinence, and speech impediments) and/or generalized neurological symptoms and signs (e.g., headache, nausea, vomiting, dizziness, sleep-wake disturbances, and mental-status abnormalities) [30,31]. Focal neurological symptoms and signs are provoked by the compression or destruction of nearby normal tissues and might give a very specific hint about the presence of an intracranial tumor and its location. These symptoms develop in many patients with primary or metastatic intracranial tumors and usually have sub-acute and progressive development [31]. On the other hand, generalized symptoms reflect increased intracranial pressure caused by the tumor’s mass effect, edema and disruption of the blood-brain barrier, causing leakage of water, electrolytes, and proteins into neural tissue [31,32]. 

Although focal neurological symptoms represent the most conventional symptoms for brain tumors, psychiatric symptoms are not uncommon, with a prevalence ranging from 50% [33] to 90% [34] in different studies. In our review, 17 out of 165 cases (10.3%) of the cases had exclusively psychiatric symptoms and 63 cases (38.2%) psychiatric and generalized neurological symptoms without focal neurological symptoms. These findings indicate that brain tumors can occur with psychiatric symptoms only, in the absence of neurological signs, or even in the “retarded” presence of neurological signs, making the diagnosis of brain tumors much more challenging, and there may be a long latent period before the organic pathology is identified. Indeed, we observed the appearance of either focal or generalized neurological symptoms after psychiatric symptoms to be associated with a significantly higher delay in the diagnosis of brain tumors in adults (Table 2 and Table 3). However, it cannot also be excluded that the generally long delay in the diagnosis we observed in our data might be due to the fact that case reports often involve particularly challenging cases. Therefore, these results might not be generalizable to the whole population of patients with brain tumors in which psychiatric symptoms appeared before neurologic manifestations.

It is also important to note that, regardless of the treatment approach, the psychiatric symptoms reversed in 93 out of 100 cases after the surgical removal of the tumor or its management through chemotherapy and radiotherapy, suggesting that early diagnosis of brain tumors is also crucial for treatment of the accompanying psychiatric symptoms.

### 4.1. Factors Affecting the Clinical Presentation of Brain Tumors

Although any psychiatric symptom or constellation of symptoms can occur, a number of factors such as rate of growth, location, tumor type and intracranial pressure may affect the clinical presentation in patients with brain tumors. A rapidly growing tumor will more likely cause symptoms, while slow-growing tumors, such as first grades of meningioma, allow for more adaptive changes of the brain, and therefore, are more likely to be “neurologically silent” and may present only with psychiatric symptoms without or with retarded appearance of neurological signs, as in some of our included cases [27,28,35]. However, we only observed marginally significant differences in tumor types between cases in which focal neurological symptoms appeared along with rather than after psychiatric symptoms and did not observe increased frequency of meningiomas in the second group. The location of the disrupted brain tissue might also determine the nature of the associated symptoms. While we did not observe significant differences among cases included in our review, possibly due to a relatively limited number of included cases, psychiatric symptoms have been suggested to be more common among patients with supratentorial compared to infratentorial tumors [12,36]. Frontal lobe tumors are reported to manifest with personality changes and impaired judgment, while tumors in the temporolimbic areas can induce auditory and visual hallucinations, mania, panic attacks, or amnesia [37]. Also, tumors in the frontal and temporal lobes have been suggested to cause more psychiatric symptoms than those in parietal or occipital lobes. More specifically, tumors in the left-sided frontal lobes have been suggested to be associated with depression, while those in the right-sided frontal lobes may be associated with manic features [38]. In addition, a significant association has been found between anorexia and hypothalamic tumors, whereas a probable association has been suggested between psychotic symptoms and pituitary tumors, or between memory symptoms and thalamic tumors [39]. However, a meta-analysis by Madhusoodanan et al. [40] suggested that tumor location is not always associated with specific symptoms. Some studies tried to relate certain brain tumor types with specific symptoms, with contrasting results. In fact, the relationship between brain tumor type and symptoms may be only as a function of location, duration and rate of growth. For instance, meningiomas are most commonly found in the frontal region of the brain but are slower growing than gliomas. Therefore, patients with meningiomas in the frontal region may be more likely to present with psychiatric symptoms and generalized neurological symptoms [27,28,41,42,43,44], while tumors that cause multiple lesions are more likely to cause more serious focal symptoms than single tumors [45,46,47,48,49]. Finally, the raised intracranial pressure from the tumor may increase appearance of psychiatric symptoms [50]. Because of the absence of a lymphatic system in the brain, fluid accumulation from leaking capillaries cannot be removed easily except by slow diffusion towards the cerebrospinal pathways [50]. Some studies hypothesized that the severity of symptoms may be related to the edema caused by the tumor in the surrounding brain tissue, which may result in disruption in the dynamics of intracerebral circulation or even obstruction of the ventricular system, rather than to the actual size and volume of the tumor mass [50,51]. The combined presence of all these factors may affect the nature, course and intensity of symptoms among cases.

### 4.2. Which Symptoms and Signs Must Raise Suspicion about A Possible Brain Tumor?

It is challenging to suspect a brain tumor when patients with psychiatric symptoms have a normal neurological examination. In a high number of cases, the presentation can be highly aspecific, as suggested by a recent study that did not find indicators able to distinguish glioma patients based on prediagnostic symptoms in the 5 years prior to diagnosis [52].

It is important to note that our case reports have been recorded since 1970s. During that era, the diagnosis of brain tumors was much more challenging since the first CT scans were introduced in 1971, and the first human MRI images in 1977 [53]. Nowadays, advanced neuro-imaging techniques that play a crucial role in the surveillance and diagnosis of brain tumors are available [54]. However, it was reported that patients see their primary care doctor on average three or more times before brain tumor diagnosis [55]. Patients with high-risk symptoms such as focal neurological symptoms are usually diagnosed quickly, but patients with lower-risk and less specific symptoms such as psychiatric or generalized neurological symptoms are more challenging to diagnose [56]. Brain imaging in psychiatric disorders is not systematically carried out due to the rarity of psychiatric presentation in the absence of neurological manifestations in brain tumor cases. However, despite the lack of consensus guidelines concerning its performance currently, it should be carefully considered. Special attention should be focused toward any sudden or late acute change in behavior or cognitive abilities, especially in patients without a personal or a family history of psychiatric illnesses [57], and in a context of an exaggeration of personality characteristics or a reversal of habitual traits (e.g., apathy, hygiene, social withdrawal) [47,58]. In addition, in a number of cases included in our review brain tumors caused treatment-resistant psychiatric syndromes which were resolved or at least improved after surgery [37,42,59]. Attention should also be focused on “atypical” psychiatric features. For instance, a large proportion of the reported cases of anorexia nervosa in patients with brain tumors lacked the evidence of “typical” anorectic behavior toward self-starvation [60,61,62]. The diagnosis of anorexia nervosa in our literature cases was based on criteria such as eating very small quantities of food, fear of becoming obese or retarded puberty and growth [60,63,64]. We found that some aspects should have led to early differential diagnostic considerations. Firstly, anorexia nervosa occurs more frequently in females and is rarer in males or children, while brain tumors may be found in both sexes; secondly, patients suffering from anorexia nervosa are usually hyperactive, whereas patients with brain tumors may become extremely tired, weak, and lethargic [65]; thirdly, in most of our cases there was no evidence of distorted body image, “self-induced” vomiting, or physical signs such as lanugo hair, bradycardia, dry skin, and hypothermia [66]; fourthly, in some cases the patients expressed their desire to eat food and re-gain the weight they had lost, but complained from swallowing difficulties [60,64]. The patient realized that his/her emaciated state and bizarre eating habits were abnormal, which is not the case of anorexia nervosa patients who refuse to eat or have bizarre food preferences, as a result of their perception of their appearance; fifthly; the onset of anorexia nervosa is commonly at puberty or prepuberty, often just before or at menarche, while cases of brain tumors can occur at any age [67,68]. Consequently, while only few cases fulfill the DSM criteria for anorexia nervosa [69], the majority can be characterized as “atypical anorexia nervosa”. This represents an example of how atypical psychiatric symptoms can mask the presence of a brain tumor. Even when the diagnosis of anorexia nervosa seems certain, it should be diagnosed only if physical illness can be excluded with a very high degree of certainty, primarily by means of a careful medical history, thorough physical and anthropometric examinations, endocrine assessment, as well as neuroimaging, particularly if headache and vomiting are intermittently present. In pediatric cases, we found eating disorders to be associated with higher delay in brain tumor diagnosis in unadjusted analyses. However, conclusive evidence cannot be drawn due to the low number of pediatric cases included in our review.

Another important point is which non-focal neurological symptoms and signs must raise suspicion about a possible brain tumor. In adults, we found that cognitive deficits were more frequent in patients in whom focal neurological symptoms appeared along with rather than after psychiatric symptoms. Consistently, cognitive symptoms were negatively associated with a delay in brain tumor diagnosis, suggesting that these symptoms often lead to diagnosis of the underlying condition. Cognitive deficits can manifest as impairments of executive function, memory, learning, attention, language, person-time-space orientation, and visuo-spatial abilities. They are among the most common neurological symptoms in patients with brain tumors and were observed in 65 (39.4%) of all 165 cases included in our systematic review. Factors suggested to be associated with cognitive dysfunction include tumor location [70], tumor size, edema, and tumor type. In slow-growing tumors, compensation and substitutive neural mechanisms tend to mask focal deficits, and provoke only mild cognitive deficits at disease onset, while in high-grade tumors, patients may present confusion, headache, and physical symptoms, and the cognitive decline may be more pronounced [71]. As suggested by Douw et al. [72], brain tumors not only may cause focal neuron disruption and mass effects, but also alterations of brain connectivity. Pathological changes in amplitude and synchronization of low-frequency connectivity, involving different neural networks, have been related to learning and memory deficits [73]. Together with toxic and metabolic insults, such alterations explain the non-focal cognitive patterns of brain tumors, suggesting whole brain dysfunction.

Headache, which was reported in 59 cases (35.8%) in this review, is another non-specific important symptom which may point into the direction of an organic pathology. Headache is frequently the initial symptom in several cases and is not a common complaint in all psychiatric disorders (e.g., psychosis) [74]. The appearance of a headache that becomes persistent [75], progressively worsening [76], localized [77], occurring with awakening or nocturnal [57], associated with or relieved by emesis [78], resistant to analgesics [79], must lead to investigate the possible presence of a brain tumor. Besides, Soniat [80] warned that severe headaches quickly relieved by changing the position of the head, and episodes of hypersomnia, are common in brain tumors and are often mistaken for conversion symptoms. The relevance of headache as a potential warning sign has also been underlined by a recent case-control study reporting that it was experienced by 39% of patients with glioma in the year prior to diagnosis [81].

Sleep-wake disturbances are defined as perceived or actual alterations in sleep that result in impaired daytime functioning. While they are frequent in patients with psychiatric disorders such as anxiety, depression, and bipolar disorder [82,83,84], they were also frequent among cases included in our review, being reported in 46 (27.9%) of the included cases. We found sleep disturbances to be more frequent in cases in which neurological symptoms appeared along with rather than after psychiatric symptoms (Table 2 and Table 3). However, no association with delay in brain tumor diagnosis was observed. Tumors located near the neural substrates that regulate sleep-wake rhythms and hormonal secretions may disrupt the patient’s sleep patterns or quality. Accordingly, in adults we observed sleep disturbances to be more frequent in patients with supratentorial compared to infratentorial tumors. However, other studies did not observe a direct relationship between tumor’s location and sleep disturbances [85,86,87]. As in the case of other neurological illnesses, sleep disturbances may also accompany other symptoms such as fatigue, depression, and cognitive impairment in the patients with brain tumors, and be associated with disease progression [30,88,89].

Nausea and vomiting occurred in 37 (22.4%) of literature cases before diagnosis but also, more interestingly, in 16 cases (48.5%) out of 33 children cases compared to 19 cases (17.6%) and two cases (8.3%) out of 108 adult and 24 older adult cases respectively (*p* < 0.0001). Vomiting is common in children [90] and can be caused by several conditions such as gastroenteritis or airway and urinary tract infections. However, it can also be caused by intracranial pathology both through increased intracranial pressure and direct stimulation of the vomiting center in the brainstem. Since vomiting is often caused by gastrointestinal disorders, it can be overlooked in the absence of focal neurological symptoms. Accompanying symptoms, such as fever, diarrhea, cough or weight loss, might guide the diagnosis. In several cases the repetitive and effortless vomiting did not induce water and electrolytes disturbances, despite the chronic duration and severity of vomiting [91], a situation that does not match a gastrointestinal cause for vomiting. Although it is not a specific neurological sign, and the differential diagnosis of vomiting is extensive, intracerebral causes should always be considered, especially if intractable or chronic vomiting are present, if the water and electrolyte balance and the acid base balance are not disturbed, or if the vomiting is associated with headaches [12,92].

Findings from our review have to be interpreted in light of several limitations. Firstly, our review was focused on a specific subgroup of patients presenting psychiatric symptoms before or along with the diagnosis of an underlying brain tumor. In addition, case reports usually involve particularly challenging cases or cases of educational value. Due to these aspects, as well as to the rarity of psychiatric symptoms in the absence of neurological manifestations in patients with brain tumors, our systematic search of case reports retrieved a limited number of cases, leading to our analyses to be underpowered to fully delineate the profile of psychiatric symptoms in cases with underlying brain tumors, especially in the pediatric and older adult groups. Secondly, case reports have a retrospective design, and the description of symptoms was not uniform over the years. Potential classes of psychiatric and neurological symptoms may not be included completely, and standardized scales or quantitative measures of symptoms were not available. Thirdly, since most cases did not report information regarding the specific histology or behavior according to the WHO classification, it was not possible to analyze differences between benign and malignant tumors. Finally, since case reports are not chosen from representative population samples, their results cannot be generalized to the whole population of patients with brain tumors. Nevertheless, their systematic summary and analysis can provide insights into the psychiatric and neurological manifestations caused by brain tumors before diagnosis, to generate hypotheses that might be worth of investigation in larger and possibly prospective studies. 

## 5. Conclusions

The early detection of brain tumors is essential, as these disorders can exert severe consequences and may be life-threatening. Early diagnosis and treatment can reduce or alleviate the associated psychiatric and neurological symptoms. In most of the included cases, psychiatric and minor neurological symptoms preceded frank focal neurological signs by months or even years, suggesting that psychiatric manifestations can present not only as symptoms of brain tumors, but as an anticipatory sign. In these cases, brain imaging and detailed mental state testing, in addition to the traditional neurological examination and neuropsychological assessment, may be of help to reduce the delay in brain tumor diagnosis. Brain tumors are highly challenging. Psychiatric manifestation as a potential indicator for the disease can present the “tip of the iceberg” that we should be aware of to ensure timely diagnosis of brain tumors and to improve the prognosis of these patients.

## Figures and Tables

**Table 1:** Clinical characteristics of 165 brain tumor cases with psychiatric symptoms.

Variable	*n* (%)
Gender Male Female	165 77 (46.7) 88 (53.3)
Age Pediatric (< 18 years) Adult (18 ≤ age <65) Older Adult (≥ 65 years)	165 33 (20%) 108 (65.5%) 24 (14.5%)
Tumor Location Frontal Temporal Parietal Occipital Parieto-occipital Parieto-temporal Parieto-frontal Fronto-temporal Ventricular Posterior Fossa Pineal Suprasellar Parasellar Hypothalamus Thalamus Corpus Callosum Basal Ganglia Cingulated area Multi-localized	163 35 (21.2%) 23 (13.9%) 4 (2.4%) 4 (2.4%) 3 (1.8%) 5 (3%) 6 (3.6%) 5 (3%) 19 (11.5%) 22 (13.3%) 7 (4.2%) 8 (4.8%) 1 (0.6%) 4 (2.4%) 2 (1.2%) 3 (1.8%) 1 (0.6%) 1 (0.6%) 10 (6.1%)
Tumor Type Categorization Diffuse astrocytic and oligodendroglial tumors Meningiomas Germ cell tumors Mesenchymal, non-meningothelial tumors Tumors of the sellar region Neuronal and mixed neuronal glial tumors Ependymal tumors Choroid plexus tumors Tumors of the pineal region Embryonal tumors Other astrocytic tumors Other gliomas	165 48 (29.1%) 54 (32.7%) 12 (7.3%) 11 (6.7%) 9 (5.5%) 10 (6.1%) 4 (2.4%) 4 (2.4%) 2 (1.2%) 1 (0.6%) 8 (4.8%) 2 (1.2%)

Tumor types were categorized according to the World Health Organization (WHO) Classification of tumors of the central nervous system (CNS), 4th ed. [17].

**Table 2:** Characteristics of cases in which generalized neurological symptoms appeared along with or after psychiatric symptoms.

	Pediatric Cases				Adult Cases				Older Adult Cases			
Variable	Generalized Neurological Symptoms along with Psychiatric Symptoms (*n* = 18)	Generalized Neurological Symptoms after Psychiatric Symptoms (*n* = 9)	Stat	*p*	Generalized Neurological Symptoms along with Psychiatric Symptoms (*n* = 39)	Generalized Neurological Symptoms after Psychiatric Symptoms (*n* = 40)	Stat	*p*	Generalized Neurological Symptoms along with Psychiatric Symptoms (*n* = 18)	Generalized Neurological Symptoms after Psychiatric Symptoms (*n* = 5)	Stat	*p*
Age, years ^a^	10 (8, 14)	14 (11, 16)	**42.5**	**0.046**	50 (35, 55)	42 (32, 53)	699.5	0.43	68 (66, 74)	69 (66, 75)	41.5	0.80
Gender, *n* (%F)	7 (38.9 %)	4 (44.4%)	0.08	1.00	23 (59.0%)	20 (50.0%)	0.64	0.50	11 (61.1%)	4 (80.0%)	0.62	0.62
Type of tumor (*n*, % of the three most frequent types) **^b^**	Diffuse astrocytic and oligodendroglial (7, 39%); meningiomas (6, 33%); mesenchimal (2, 11%);	Diffuse astrocytic and oligodendroglial (4, 44%); meningiomas (3, 33%); mesenchimal (1, 11%)	1.30	0.94	Meningiomas (10, 26%); diffuse astrocytic and oligodendroglial (7, 18%); tumors of the sellar region (6, 15%)	Diffuse astrocytic and oligodendroglial (11, 28%); meningiomas (8, 20%), germ cell (7, 18%)	18.07	0.05	Meningiomas (10, 56%); diffuse astrocytic and oligodendroglial (7, 39%); mesenchimal (1, 6%)	Meningiomas (3, 60%); diffuse astrocytic and oligodendroglial (2, 40%)	0.29	0.86
Location, *n* (% infratentorial) **^c^**	4 (25%)	2 (25%)	0.00	1.00	4 (10%)	10 (25%)	2.94	0.14	3 (17%)	0 (0%)	1.02	1.00
Time from onset of symptoms to diagnosis, months **^a^**	12 (4, 30)	14 (3, 30)	76.5	1.00	5 (2, 18)	24 (7, 60)	**381.5**	**0.002**	4 (2, 8)	6 (6, 78)	18.00	0.08
Resolution of psychiatric symptoms after tumor treatment, *n* (%)	15 (94%) ^d^	4 (80%) ^d^	0.84	0.43	17 (85%) ^e^	28 (93%) ^e^	0.93	0.38	4 (100%) ^f^	3 (100%) ^f^	- ^f^	- ^f^
Psychiatric Symptoms												
Anxiety, *n* (%)	7 (39%)	0 (0%)	4.73	0.06	10 (26%)	6 (15%)	1.38	0.27	3 (17%)	3 (60%)	3.81	0.09
Apathy, *n* (%)	0 (0%)	1 (11%)	2.08	0.33	3 (8%)	7 (18%)	1.72	0.31	7 (39%)	1 (20%)	0.62	0.62
Depression, *n* (%)	4 (22%)	4 (44%)	1.42	0.38	19 (49%)	13 (33%)	2.16	0.17	4 (22%)	2 (40%)	0.64	0.58
Eating disorder, *n* (%)	10 (56%)	5 (56%)	0.00	1.00	3 (8%)	4 (10%)	0.13	1.00	0 (0%)	0 (0%)	-	-
Manic symptoms, *n* (%)	2 (11%)	0 (0%)	1.08	0.54	1 (3%)	6 (15%)	3.78	0.11	0 (0%)	0 (0%)	-	-
Miscellaneous, *n* (%)	3 (17%)	0 (0%)	1.69	0.53	2 (5%)	3 (3%)	0.19	1.00	1 (6%)	0 (0%)	0.29	1.00
Personality changes, *n* (%)	2 (11%)	1 (11%)	0.00	1.00	10 (26%)	10 (26%)	0.00	1.00	6 (33%)	2 (40%)	0.08	1.00
Psychotic symptoms, *n* (%)	4 (22%)	4 (44%)	1.42	0.38	16 (41%)	15 (36%)	0.10	0.82	5 (28%)	2 (40%)	0.28	0.62
Neurological Symptoms												
Cognitive deficits, *n* (%)	3 (17%)	5 (56%)	4.35	0.07	26 (67%)	15 (38%)	**6.73**	**0.013**	11 (31%)	5 (100%)	2.80	0.27
Delayed puberty, *n* (%)	2 (11%)	0 (0%)	1.08	0.54	0 (0%)	0 (0%)	-	-	0 (0%)	0 (0%)	-	-
Dizziness, *n* (%)	1 (6%)	2 (22%)	1.69	0.25	4 (10%)	8 (20%)	1.46	0.35	3 (17%)	2 (40%)	1.25	0.29
Growth retardation, *n* (%), *n* (%)	5 (28%)	0 (0%)	3.07	0.14	0 (0%)	0 (0%)	-	-	0 (0%)	0 (0%)	-	-
Headache, *n* (%)	7 (39%)	2 (22%)	0.75	0.67	18 (46%)	23 (58%)	1.02	0.37	6 (33%)	2 (40%)	0.08	1.00
Motor deficits, *n* (%)	8 (44%)	5 (56%)	0.30	0.70	17 (44%)	16 (40%)	0.11	0.82	8 (44%)	2 (40%)	0.03	1.00
Nausea/Vomiting, *n* (%)	11 (61%)	5 (56%)	0.08	1.00	8 (21%)	11 (28%)	0.53	0.60	2 (11%)	0 (0%)	0.61	1.00
Ocular impairments, *n* (%)	5 (28%)	3 (33%)	0.09	1.00	13 (33%)	7 (118%)	2.62	0.13	0 (0%)	2 (40%)	**7.89**	**0.04**
Seizures, *n* (%)	2 (11%)	0 (0%)	1.08	0.54	5 (13%)	6 (15%)	0.08	1.00	0 (0%)	2 (40%)	**7.89**	**0.04**
Sleep disturbances, *n* (%)	5 (28%)	4 (44%)	0.75	0.42	21 (54%)	11 (28%)	**5.69**	**0.022**	4 (22%)	1 (20%)	0.01	1.00
Speech impediments, *n* (%)	1 (6%)	1 (11%)	0.27	1.00	5 (13%)	9 (23%)	1.27	0.38	2 (11%)	1 (20%)	0.27	0.54
Urinary incontinence, *n* (%)	0 (0%)	0 (0%)	-	-	2 (5%)	6 (15%)	2.23	0.26	3 (17%)	0 (0%)	0.96	1.00

Cases were stratified according to age at diagnosis (pediatric cases: age < 18 years; adult cases: age between 18 and 64 years; and older adult cases: age ≥ 65 years). Age at diagnosis and months from onset of psychiatric symptoms to diagnosis were compared between patients with neurological symptoms appeared along with vs. appeared after psychiatric symptoms using Mann Whitney U test. All the other variables were compared between the two groups using Fisher’s exact test. Significant results are reported in bold. ^a^ = median (25, 75 percentile); ^b^ Analyses conducted on all available types of tumor. Only the three most frequent types are reported in the table; ^c^ Tumors were classified in supratentorial and infratentorial. As only two pediatric cases shower both supra and infratemporal locations, these cases were excluded from analyses. For pediatric cases, data only available for 16 and nine participants for which generalized neurological symptoms appeared along with and after psychiatric symptoms, respectively; ^d^ Data only available for 16 and five participants for which generalized neurological symptoms appeared along with and after psychiatric symptoms, respectively; ^e^ Data only available for 20 and 30 participants for which generalized neurological symptoms appeared along with and after psychiatric symptoms, respectively; ^f^ Data only available for five and three participants for which generalized neurological symptoms appeared along with and after psychiatric symptoms, respectively. Abbreviations: F, female; Stat, statistics.

**Table 3:** Characteristics of cases in which focal neurological symptoms appeared along with or after psychiatric symptoms.

	Pediatric cases				Adult Cases				Older Adult Cases			
Variable	Focal Neurological Symptoms along with Psychiatric Symptoms (*n* = 4)	Focal Neurological Symptoms after Psychiatric Symptoms (*n* = 19)	Stat	*p*	Focal Neurological Symptoms along with Psychiatric Symptoms (*n* = 16)	Focal Neurological Symptoms after Psychiatric Symptoms (*n* = 49)	Stat	*p*	Focal Neurological Symptoms along with Psychiatric Symptoms (*n* = 6)	Focal Neurological Symptoms after Psychiatric Symptoms (*n* = 8)	Stat	*p*
Age, years ^a^	8 (4, 14)	13 (10, 15)	22.00	0.22	49 (30, 59)	41 (33, 55)	361.0	0.64	72 (68, 79)	68 (65, 73)	14.00	0.23
Gender, *n* (%F)	1 (25%)	7 (37%)	0.20	1.00	11 (69%)	26 (57%)	1.21	0.39	2 (33%)	5 (63%)	1.17	0.59
Type of tumor (*n*, % of the three most frequent types) **^b^**	Diffuse astrocytic and oligodendroglial (2, 50%); mesenchimal (1, 25%); germ cell (1, 25%)	Meningiomas (9, 47%); diffuse astrocytic and oligodendroglial (6, 32%); mesenchimal (2, 11%)	7.92	0.10	Meningiomas (5, 27%); diffuse astrocytic and oligodendroglial (4, 27%); tumors of the sellar region (2, 13%)	Meningiomas (14, 29%); diffuse astrocytic and oligodendroglial (13, 27%); neuronal and mixed neuronal glia (6, 12%)	5.49	0.70	Meningiomas (5, 83%); diffuse astrocytic and oligodendroglial (1, 17%)	Meningiomas (4, 50%); diffuse astrocytic and oligodendroglial (4, 50%)	1.66	0.30
Location, *n* (% infratentorial) **^c^**	1 (25%)	6 (38%)	0.15	1.00	1 (7%)	9 (18%)	1.36	0.43	1 (17%)	2 (25%)	0.14	1.00
Time from onset of symptoms to diagnosis, months **^a^**	4 (1, 8)	13 (4, 36)	15.00	0.07	3 (1, 18) ^d^	18 (6, 48) ^d^	**161.5**	**0.012**	4 (1, 68)	18 (5, 57)	11.00	0.31
Resolution of psychiatric symptoms after tumor treatment, *n* (%)	4 (100%) **^e^**	13 (100%) **^e^**	-	-	7 (86%) **^f^**	28 (90%) **^f^**	0.06	1.00	1 (100%) **^g^**	2 (100%) **^g^**	-	-
Psychiatric Symptoms												
Anxiety, *n* (%)	1 (25%)	5 (26%)	0.00	1.00	2 (13%)	10 (20%)	0.50	0.71	0 (0%)	4 (50%)	4.20	0.09
Apathy, *n* (%)	0 (0%)	1 (5%)	0.22	1.00	0 (0%)	9 (18%)	3.41	0.10	2 (33%)	2 (25%)	0.12	1.00
Depression, *n* (%)	0 (0%)	7 (37%)	2.12	0.27	5 (31%)	13 (27%)	0.13	0.75	0 (0%)	3 (38%)	2.86	0.21
Eating disorder, *n* (%)	0 (0%)	10 (53%)	3.73	0.10	0 (0%)	5 (10%)	1.77	0.32	0 (0%)	0 (0%)	-	-
Manic Symptoms, *n* (%)	0 (0%)	1 (5%)	0.22	1.00	1 (6%)	5 (10%)	0.23	1.00	0 (0%)	0 (0%)	-	-
Miscellaneous, *n* (%)	2 (50%)	3 (16%)	2.27	0.19	3 (19%)	3 (6%)	2.30	0.15	2 (33%)	0 (0%)	3.11	0.17
Personality changes, *n* (%)	1 (25%)	3 (16%)	0.20	1.00	3 (19%)	14 (29%)	0.60	0.53	3 (50%)	2 (25%)	0.93	0.58
Psychotic symptoms, *n* (%)	2 (50%)	5 (26%)	0.88	0.56	10 (63%)	21 (43%)	1.87	0.25	1 (17%)	2 (25%)	0.14	1.00
Neurological Symptoms												
Cognitive deficits, *n* (%)	0 (0%)	5 (26%)	1.35	0.54	9 (56%)	16 (33%)	2.84	0.14	3 (50%)	6 (75%)	0.93	0.58
Delayed puberty, *n* (%)	0 (0%)	2 (11%)	0.46	1.00	0 (0%)	0 (0%)	-	-	0 (0%)	0 (0%)	-	-
Dizziness, *n* (%)	0 (0%)	3 (16%)	0.73	1.00	1 (6%)	5 (10%)	0.22	1.00	0 (0%)	3 (38%)	2.86	0.21
Growth retardation, *n* (%)	0 (0%)	4 (21%)	1.02	1.00	0 (0%)	0 (0%)	-	-	0 (0%)	0 (0%)	-	-
Headache, *n* (%)	2 (50%)	4 (21%)	1.44	0.27	5 (31%)	26 (53%)	2.30	0.16	3 (50%)	3 (38%)	0.22	1.00
Motor deficits, *n* (%)	2 (50%)	15 (79%)	1.44	0.27	13 (81%)	23 (47%)	**5.75**	**0.02**	5 (83%)	6 (75%)	0.14	1.00
Nausea/Vomiting, *n* (%)	2 (50%)	10 (53%)	0.01	1.00	2 (13%)	11 (22%)	0.75	0.49	0 (0%)	2 (25%)	1.75	0.47
Ocular impairments, *n* (%)	2 (50%)	7 (37%)	0.24	1.00	6 (38%)	15 (31%)	0.26	0.76	1 (17%)	2 (25%)	0.14	1.00
Seizures, *n* (%)	2 (50%)	0 (0%)	**10.41**	**0.024**	5 (31%)	13 (27%)	0.13	0.75	0 (0%)	2 (25%)	1.75	0.47
Sleep disturbances, *n* (%)	3 (75%)	3 (16%)	**6.01**	**0.04**	5 (31%)	14 (29%)	0.04	1.00	1 (17%)	0 (0%)	1.44	0.43
Speech impediments, *n* (%)	0 (0%)	2 (11%)	0.46	1.00	4 (25%)	14 (29%)	0.07	1.00	1 (17%)	2 (25%)	0.14	1.00
Urinary incontinence, *n* (%)	0 (0%)	0 (0%)	-	-	1 (6%)	8 (17%) ^h^	1.08	0.43	2 (33%)	1 (13%)	0.88	0.54

Cases were stratified according to age at diagnosis (pediatric cases: age < 18 years; adult cases: age between 18 and 64 years; and older adult cases: age ≥ 65 years). Age at diagnosis and months from onset of psychiatric symptoms to diagnosis were compared between patients with neurological symptoms appeared along with vs. appeared after psychiatric symptoms using Mann Whitney U test. All the other variables were compared between the two groups using Fisher’s exact test. Significant results are reported in bold. ^a^ = median (25, 75 percentile); ^b^ Analyses conducted on all available types of tumor. Only the three most frequent types are reported in the table; ^c^ Tumors were classified in supratentorial and infratentorial. As only two pediatric cases shower both supra and infratemporal locations, these cases were excluded from analyses; ^d^ Data only available for 13 and 46 participants for which focal neurological symptoms appeared along with and after psychiatric symptoms, respectively; ^e^ Data only available for four and 13 participants for which focal neurological symptoms appeared along with and after psychiatric symptoms, respectively; ^f^ Data only available for eight and 31 participants for which focal neurological symptoms appeared along with and after psychiatric symptoms, respectively; ^g^ Data only available for one and two participants for which focal neurological symptoms appeared along with and after psychiatric symptoms, respectively; ^h^ Data available for 48 patients. Abbreviations: F, female; Stat, statistics.

**Table 4:** Association between clinical characteristics and time from onset of symptoms to diagnosis.

	Pediatric Cases (*n* = 32)		Adult Cases (*n* = 100)		Older Adult Cases (*n* = 22)	
Variable	Statistics	*p*	Statistics	*p*	Statistics	*p*
Age at diagnosis	0.2	0.21	**−0.2**	**0.04**	0.2	0.42
Female gender	87.5	0.17	1224.0	0.90	32.5	0.11
Type of tumor	3.4	0.64	9.5	0.58	1.5	0.46
Tumor location	82.0	0.94	584.5	0.41	4.5	0.09
Psychiatric Symptoms						
Anxiety	94.5	0.95	665.5	0.25	25.5	0.19
Apathy	6.5	0.44	555.5	0.27	44.0	0.58
Depression	71.5	0.18	892.0	0.19	40.5	0.59
Eating disorders	**63.5**	**0.014**	**246.5**	**0.007**	-	-
Manic symptoms	26.0	0.79	327.5	0.61	-	-
Miscellaneous symptoms	66.5	0.96	368.5	0.62	4.0	0.08
Personality changes	**19.0**	**0.009**	893.0	0.88	43.5	0.54
Psychotic symptoms	98.0	0.65	1200.5	0.95	36.5	0.27
Neurological symptoms						
Cognitive deficits	69.0	0.25	**619.5**	**<0.0001**	51.0	0.95
Delayed puberty	1.5	0.40	-	-	-	-
Dizziness	42.0	0.95	470.0	0.83	22.5	0.26
Growth retardation	40.0	0.17	-	-	-	-
Headache	95.0	0.74	1141.5	0.73	47.0	0.57
Motor deficits	117.5	0.71	1017.5	0.60	53.5	0.74
Nausea/Vomiting	104.5	0.39	558.0	0.11	4.5	0.46
Ocular Impairments	89.0	0.56	743.0	0.62	**7.0**	**0.04**
Seizures	15.0	0.29	612.5	0.44	**2.0**	**0.04**
Sleep disturbances	59.0	0.06	975.0	0.48	21.0	0.10
Speech impediments	27.5	0.85	613.0	0.26	11.0	0.11
Urinary incontinence	-	-	324.5	0.61	26.0	0.86

Cases were stratified according to age at diagnosis (pediatric cases: age < 18 years; adult cases: age between 18 and 64 years; and older adult cases: age ≥ 65 years). The association between time from onset of symptoms to diagnosis and (1) age at diagnosis was analyzed using Spearman’s correlation analysis, (2) type of tumor using Kruskal-Wallis test, and (3) all the other variables using Mann-Whitney U test. Significant results are reported in bold.

## Data Availability

The data presented in this review are available in the tables or Supplementary Materials.

## Supplementary Materials

The following are available online at https://www.mdpi.com/2076-3425/11/3/301/s1, Table S1: Characteristics of “pediatric group” case reports with initial psychiatric symptoms with or without generalized and/or neurological signs and symptoms; Table S2: Characteristics of “Adults group” case reports with initial psychiatric symptoms with or without generalized and/or neurological signs and symptoms; Table S3: Characteristics of “Older Adults group” case reports with initial psychiatric symptoms with or without generalized and/or neurological signs and symptoms; Table S4: Frequencies of psychiatric and neurological symptoms occurring before the diagnosis of brain tumor in the retrieved case reports according to age groups; Table S5: Frequencies of psychiatric or neurological symptoms according to tumor location; Table S6: Frequencies of the case reports with initial psychiatric symptoms with or without generalized and/or neurological signs and symptoms, according to age groups.
