# Supplementary material for: Presenting Psychiatric and Neurological Symptoms and Signs of Brain Tumors before Diagnosis: A Systematic Review
Source: Brain Sci. 2021 Feb 27;11(3):301. doi: 10.3390/brainsci11030301 (PMC7997443; doi:10.3390/brainsci11030301)
Supplement: Supplementary file 1 [file brainsci-11-00301-s001.pdf]

brain sciences

MDPI

Review

Presenting Psychiatric and Neurological Symptoms and Signs of Brain Tumors before Diagnosis: A Systematic Review

Fatima Ghandour [1,2], Alessio Squassina [1], Racha Karaky [3], Mona Diab-Assaf [2], Paola Fadda [1,4,5,6,*] and Claudia Pisanu [1]

[1] Department of Biomedical Sciences, Division of Neuroscience and Clinical pharmacology, University of Cagliari, 09042 Monserrato, Italy; f.ghandour@studenti.unica.it (F.G.); squassina@unica.it (A.S.); claudia.pisanu@unica.it (C.P.)
[2] EDST, Pharmacology and Cancerology Laboratory, Faculty of Sciences, Lebanese University, Beirut 1500, Lebanon; mdiabassaf@ul.edu.lb
[3] Drug-Related Sciences department, Faculty of Pharmacy, Lebanese University, Hadath 1500, Lebanon; racha.karaky@ul.edu.lb
[4] Centre of Excellence "Neurobiology of Addiction", University of Cagliari, 09042 Monserrato, Italy
[5] CNR Institute of Neuroscience - Cagliari, National Research Council, 09042 Monserrato, Italy
[6] National Institute of Neuroscience (INN), 10126 Turin, Italy
* Correspondence: pfadda@unica.it

Table S1. Characteristics of "pediatric group" case reports (age < 18 years) with initial psychiatric symptoms with or without generalized and/or neurological signs and symptoms.

Ref.	Age	Gender	Tumor type	Tumor location	Psychiatric symptoms (P.S)	Neurological symptoms	Time from symptoms to diagnosis	P.S after tumor treatment
[1]	5	F	Diffuse intrinsic pontine glioma	Pontine	Personality changes	Motor deficits	3 weeks	N.S.
[2]	3	M	Pilocytic Astrocytoma	Rostral medulla	Paroxysmal crying, anxiety	Nausea, vomiting, seizure	8 months	✓
[3]	11	F	Astrocytoma	Temporal	Psychosis	Cognitive deficits	3 years	N.S.
[4]	10	F	Anaplastic ependymoma	Parieto-occipital	anorexia nervosa	Headache, vomiting, dizziness, motor deficits	1 year	✓
[5]	13	F	Extraventricular neurocytoma	Cerebellum	Psychosis, miscellaneous (pathological laughter)	Motor deficits	2 years	✓
[6]	8	M	Oligodendroglioma	Left Frontal	Personality changes, miscellaneous (biting himself)	Sleep-wake disturbances, seizure	4 weeks	✓
[7]	17	M	Germinoma	Mesencephalon, mammillary tubercle, thalamus, interventricular septum, corpus callosum	Apathy, psychosis	Cognitive deficits, sleep-wake disturbances, speech impediments	3 months	N.S.
[8]	17	M	Pineal gland tumor	Pineal	Anxiety, psychosis, depression, miscellaneous (self-injurious behavior)	Cognitive deficits, motor deficits	4 years	✓
[9]	15	M	Ganglioglioma	Cingulated Area	Panic attacks	Headache	3 months	✓
[10]	13	M	Germ cell tumor	Suprasellar, Pineal	Depression	Motor deficits, ocular impairments	1 year	N.S.
[11]	15.5	F	Craniopharyngioma	Intra- and parasellar	Anorexia nervosa	Slow growth and late puberty, headache, ocular impairments	6 years	✓
[12]	5	F	Pilocytic astrocytoma	Hypothalamus, brain stem	Anorexia nervosa, anxiety, depression	Vomiting, speech impediments, motor deficits	20 months	✓
[13]	6	M	Diffuse pontine glioma	Pontine	Anxiety, personality changes, pathological laughter	Motor deficits	5 months	✓
[14]	12	M	Pinealoma	Pineal gland	Psychosis, depression, Personality changes	Headache, sleep-wake disturbances, dizziness, vomiting, ocular impairments, motor deficits	2 months	✓
[15]	9	F	Ependymoma	Brain stem	Anorexia nervosa	Vomiting	N.S.	N.S.
[16]	13	F	Ganglioglioma	Medulla oblongata	Anorexia nervosa	Sleep-wake disturbances	16 months	✓
[17]	15	F	Germinoma	Pineal gland	Psychosis	Sleep-wake disturbances, cognitive deficits	14 months	X
[18]	13	F	Meningioma	Right frontal	Anorexia nervosa		5 months	✓
[19]	13	M	Germinoma	Basal ganglia	Anxiety, psychosis, personality changes	Delayed puberty and decreased growth, motor deficits	6 months	✓
[20]	8	F	Pilocytic astrocyto-	Occipital lobe	Psychosis	Sleep-wake disturbances, headache,	6 months	✓

			ma			ocular impairments, motor deficits		
[21]	9	M	Choroid plexus papilloma	Third ventricle	Psychosis, personality changes		5 months	✓
[22]	9	M	Craniopharyngioma	Pineal gland	Anorexia nervosa, depression	Headache, vomiting, growth arrest	19 months	✓
[22]	14	M	Germinoma	Pineal gland	Anorexia nervosa	Vomiting, nausea, ocular impairments	16 months	✓
[22]	9	M	Low-grade astrocytoma	Cervicomedullary	Anorexia nervosa	Vomiting, motor deficits	3 years	N.S.
[23]	16	M	Medulloblastoma	Occipital lobe	Psychosis	Headache, vomiting, sleep-wake disturbances, ocular impairments, motor deficits	3 weeks	✓
[24]	10	F	Teratoma	Hypothalamus	Anorexia nervosa, Depression	Vomiting, cognitive deficits, motor deficits	2 months	N.S.
[25]	17	M	Teratoma	Third ventricle	Anorexia nervosa	Nausea, vomiting, headache, cognitive deficits, ocular impairments, motor deficits	13 months	N.S.
[26]	12.5	F	Germinoma	Suprasellar	Anorexia nervosa, Depression, anxiety	Headaches, vomiting, cognitive deficits, sleep-wake disturbances	2 years	X
[27]	12	M	Choroid plexus Papilloma	Fourth ventricle	Anxiety	Sleep-wake disturbances, motor deficits	4 months	✓
[28]	15	M	Teratoma	Third ventricle	Anorexia nervosa, depression	Vomiting, motor deficits, ocular impairments	2 years	✓
[29]	14	M	Polycytic astrocytoma	Brain Stem	Anorexia nervosa, depression	Dizziness, vomiting, motor deficits	4 years	✓
[30]	6.5	M	Pilocytic astrocytoma	N.S	Anorexia nervosa, mania	Vomiting	2 months	✓
[30]	10	F	Germinoma	Third ventricle	Anorexia nervosa, mania	Vomiting, ocular impairments	7 years	✓

M: Male, F: Female, N.S.: Not specified, Ref: Reference, ✓: Psychiatric symptoms resolved or improved after tumor resection/treatment, X: Psychiatric symptoms remained after tumor resection/treatment.

Table S2. Characteristics of "Adults group" case reports (age between 18 and 64 years) with initial psychiatric symptoms with or without generalized and/or neurological signs and symptoms.

Ref.	Age	Gender	Tumor type	Tumor location	Psychiatric symptoms (P.S)	Neurological symptoms	Time from symptoms to diagnosis	P.S after tumor treatment
[31]	52	F	Meningioma	Frontoparietal	Psychosis	Speech impediment, dizziness, seizure	3 weeks	✓
[32]	35	M	Glioblastoma	Right temporal	Anxiety	Cognitive deficits, headache, vomiting, motor deficits, seizure	5 months	✓
[33]	44	F	Grade I meningioma	Posterior fossa	Depression, personality changes	Headache, motor deficits, urinary incontinence	5 years	N.S.
[34]	28	F	Meningioma	Petroclival	Pathological laughter	Headache, motor deficits, Ocular impairments	5 years	✓
[35]	42	M	Craniopharyngioma	Mesencephalon	Psychosis, personality	Dizziness, cognitive deficits	6 months	N.S.

					changes			
[36]	45	F	Meningioma	Bifrontal	Depression	Cognitive deficits, sleep-wake disturbances, ocular impairments	6 months	✓
[37]	61	F	Meningoma	Suprasellar	Psychosis, personality changes, mania	Sleep-wake disturbances, speech impediment	7 months	✓
[37]	47	M	Glioblastoma	Temporoparietal	Apathy, psychosis	Headache, cognitive deficits, speech impediment, motor deficits	1.5 months	✓
[38]	42	M	Chordoid glioma	Suprasellar	Depression, psychosis, personality changes	Cognitive deficits, seizure	1 year	N.S.
[39]	28	F	Glioblastoma	Left frontal	Depression	Headache, nausea, cognitive deficits	8 weeks	✓
[40]	27	F	Central neurocytoma	Third and lateral Ventricles	Psychosis, personality changes, anxiety	Cognitive deficits, sleep-wake disturbances, motor deficits	2 months	✓
[41]	48	F	Dysembryogenic neuroepithelial tumor	Thalamus and internal capsule	Miscellaneous (self-mutilation), psychosis		3 years	N.S.
[42]	33	F	Grade I meningioma	Cerebellum	Anxiety	Headache, dizziness	2 years	✓
[43]	47	M	Ependymoma	Interventricular foramen	Depression, psychosis, personality changes	Sleep-wake disturbances	15 years	✓
[44]	38	F	Choroid plexus papilloma	Fourth ventricule	Anorexia nervosa, psychosis, anxiety	Vomiting, cognitive deficits, motor deficits	18 months	✓
[45]	61	M	Meningioma	Right frontal	Personality changes		6 months	N.S.
[46]	61	M	Glioblastoma	Frontal and corpus callosum	Personality changes, apathy	Sleep-wake disturbances, cognitive deficits	2 months	N.S.
[47]	55	F	Meningioma	Cranial fossa	Depression, anxiety	Vertigo, cognitive deficits	Few years	✓
[48]	59	M	Glioblastoma	Frontal	Depression, anxiety, Personality changes	Sleep-wake disturbances, motor deficits, urinary incontinence	1 year	N.S.
[49]	46	F	Glioblastoma	Corpus callosum	Depression, psychosis	Cognitive deficits, sleep-wake disturbances, motor deficits	2 years	N.S.
[50]	43	M	Meningioma	Right frontal	Depression	Headache, cognitive deficits, sleep-wake disturbances	2 months	N.S.
[50]	52	M	Meningioma	Bilateral frontal	Depression	Headache, cognitive deficits, sleep-wake disturbances	1 month	N.S.
[51]	54	F	Grade I meningioma	Left frontal	Depression	Cognitive deficits, sleep-wake disturbances	6 months	✓
[52]	55	M	Oligodendroglioma	Temporoparietal	Mania	Cognitive deficits	3 years	✓
[53]	21	F	Low-grade astrocytoma	Amygdala	Anxiety, psychosis		6 years	✓
[54]	60	F	Grade I meningothelial meningioma	Left frontal	Depression	Sleep-wake disturbances, headache, urinary incontinence	2 months	✓
[55]	18	F	Neuroepithelial Tumor	left temporal	Psychosis, apathy		2 years	✓
[56]	55	M	Meningioma	Right frontal	Mania, anxiety, personality changes	Vomiting, cognitive deficits, ocular impairments	2 years	✓
[57]	38	M	Choroid Plexus Pap-	Fourth ventricle	Psychosis, apathy	Intermittent headache, motor deficits, speech	2 years	✓

			illoma			impediments		
[58]	40	F	Hemangioblastoma	Fourth ventricle	Anorexia nervosa, anxiety	Headache, nausea	10 years	✓
[59]	47	F	Meningioma	Right frontal	Apathy	Nausea, cognitive deficits, sleep-wake disturbances	4.5 years	✓
[60]	25	M	Diferentiated grade II astrocytoma	Right frontal	Anorexia nervosa		2 years	✓
[61]	26	M	Meningioma	Petrous apex, temporal lobe	Depression, anxiety	Headache, nausea, vomiting, sleep-wake disturbances, motor deficits, ocular impairments	18 months	N.S.
[62]	42	M	Neuroepithelial tumour	Temporal	Anxiety	Seizure	6 years	✓
[63]	31	F	Fibrillary astrocytoma	Suprasellar	Depression, anxiety		4 months	✓
[64]	50	M	Anaplastic astrocytoma	Left temporal	Anxiety, depression	Persistent headache	7 years	✓
[65]	41	F	Meningioma	Right parietal	Psychosis	Seizure, Motor deficits	N.S.	✓
[66]	31	M	Polycystic Astrocytoma	Right frontal	Depression	Headache, cognitive deficits, sleep-wake disturbances, ocular impairments	9 months	N.S.
[67]	53	M	Glioblastoma	Frontal and temporal	Depression	Sleep-wake disturbances	10 years	N.S.
[68]	28	M	Angiocentric glioma	Temporal gyrus	Psychosis, miscellaneous (paroxysmal ictal phonemes)	Seizure, motor deficits	7 years	✓
[69]	51	M	Meningioma	Left temporal	Anxiety, depression, psychosis	Headache, cognitive deficits, sleep-wake disturbances	3 months	✓
[70]	63	M	Meningioma	Suprasellar	Apathy	Cognitive deficits, motor deficits, speech impediments	4 years	N.S.
[70]	54	F	Meningioma	Frontal	Apathy	Ocular impairments	4 months	✓
[71]	32	F	Craniopharyngioma	Third ventricle	Anorexia nervosa, depression		21 years	N.S.
[72]	60	F	Glioblastoma	Right frontal	Miscellaneous (abnormal laughter)	Motor deficits	3 months	✓
[73]	58	F	Glioblastoma	Frontoparietal	Depression, miscellaneous (crying attacks)	Sleep-wake disturbances, cognitive deficits	5 weeks	N.S.
[74]	34	M	Glioblastoma	Right frontal	Depression	Headache, cognitive deficits	6 months	✓
[75]	57	F	Oligodendroglioma	Left temporal	Psychosis	Seizure	10 years	✓
[75]	38	F	Glioblastoma	Left temporal	Psychosis	Headache, sleep-wake disturbances, motor deficits, speech impediments	5 years	N.S.
[75]	62	F	Fibrous meningioma	Fronto-temporal	Depression	Headache, sleep-wake disturbances, motor deficits	N.S.	X
[76]	19	F	Cavernous hemangioma	Sylvian valley	Anorexia nervosa, anxiety	Sleep-wake disturbances	6 years	✓
[77]	53	M	Ependymoma	Cerebellar vermis	Abnormal laughter		4 months	✓

[78]	20	M	Germinoma	Pineal gland	Anorexia nervosa, Anxiety	Vomiting, ocular impairments	18 months	✓
[79]	24	F	Glioblastoma	Left Thalamus	Depression, psychosis, personality changes	Headache, Sleep-wake disturbances, cognitive deficits, urinary incontinence, Motor deficits	> 4 years	X
[80]	18	F	Hemangioblastoma	Medulla oblongata	Anorexia nervosa	Nausea, dizziness	4 years	✓
[80]	27	F	Hemangioblastoma	Fourth ventricle	Anorexia nervosa	Headache, vomiting, motor deficits	4 years	✓
[81]	48	M	Glioblastoma	Bilateral frontal	Psychosis, Apathy		15 years	N.S.
[82]	32	F	Lipoma	Corpus callosum	Psychosis		3 years	N.S.
[83]	26	M	Central neurocytoma	Lateral ventricle	Psychosis, personality changes	Headache, cognitive deficits, ocular impairments	8 months	✓
[83]	43	M	Central neurocytoma	Lateral ventricle	Psychosis, personality changes		1.5 year	✓
[84]	24	M	Craniopharyngioma	Suprasellar	Personality changes, Miscellaneous (abnormal laughter)	Cognitive deficits, Sleep-wake disturbances, vomiting, ocular impairments	4 months	N.S.
[85]	51	F	Grade IV Aatrocytoma	Temporal	Mania, psychosis	Headache, vomiting, Dizziness, nausea, sleep-wake disturbances, speech impediments	7 months	✓
[86]	40	F	Hemangiopericytoma	Right orbitofrontal	Miscellaneous (pedophilia)	Headache, motor deficits, urinary incontinence	N.S.	✓
[87]	32	F	Craniopharyngioma	Hypothalamus	Apathy	Cognitive deficits, dizziness, headache, nausea, vomiting	1 month	X
[88]	53	F	Pineal Meningioma	Pineal region	Psychosis	Headache	2 months	✓
[89]	35	M	Meningioma	Petroclival	Miscellaneous (pathological laughter)	Motor deficits, ocular impairments	6 months	✓
[90]	33	M	Meningioma	Ventrolateral, pons and midbrain	Miscellaneous (pathological laughter)	Speech impediments, Motor deficits	4 months	✓
[91]	61	M	Glioblastoma	Right temporal	Psychosis		3 weeks	✓
[92]	28	M	Meningioma	Left petroclival	Miscellaneous (pathological laughter)	Motor deficits, ocular impairments	1 year	✓
[93]	62	M	Grade III to IV astrocytoma	Occipital & parietal	Personality changes, Mania	Nocturnal headache, Cognitive deficits, seizure, motor deficits	2 years	N.S.
[93]	26	F	Grade II astrocytoma	Frontal lobe	Anxiety	Seizure	3 years	N.S.
[94]	53	M	Grade IV astrocytoma	Left frontal	Anxiety, depression	Motor deficits	1 month	N.S.
[95]	54	F	Meningioma	Cerebellopontine	Psychosis	Cognitive deficits, seizure	9 days	N.S.
[96]	51	F	Craniopharyngioma	Hypothalamus	Depression, Personality changes	Cognitive deficits, sleep-wake disturbances	5 months	✓
[97]	56	F	Meningioma	Medial bifrontal	Apathy		3 years	✓
[97]	22	M	Grade I oligoastrocytoma	Temporal	Psychosis	Memory deficits	2 months	✓
[97]	31	M	Grade II oligoden-	Right temporal	Psychosis		N.S.	✓

			droglioma					
[97]	56	F	Glioblastoma	Bitemporal	Mania		3 months	N.S.
[98]	59	F	Meningioma	Frontal	Personality changes, apathy	Sleep-wake disturbances	4 years	N.S.
[98]	57	F	Meningioma	Corpus callosum	Personality changes, psychosis, apathy	Cognitive deficits, speech impediments	1 year	✓
[99]	35	M	Meningioma	Left frontal	Depression	Persistent headache, drowsy, vomiting, speech impediments, motor deficits, ocular impairments	5 years	✓
[99]	29	M	Meningioma	Bifrontal	Apathy	Headache, urinary incontinence, motor deficits, ocular impairments	1 year	N.S.
[100]	54	M	Meningioma	Parieto-occipital	Psychosis, personality changes, anxiety, Apathy	Seizure	27 years	N.S.
[101]	52	F	Hemangioblastoma	Cerebellum	Depression	Sleep-wake disturbance, motor deficits, urinary incontinence	2 years	N.S.
[102]	40	M	Glioblastoma	Left Temporoparietal	Depression	Headache, sleep-wake disturbances, cognitive deficits, speech impediments motor deficits, ocular impairments	3 months	N.S.
[103]	38	M	Lipoma	Sylvian fissure	Psychosis	Bifrontal headache, dizziness, sleep-wake disturbances, seizure	7 years	N.S.
[104]	58	F	Hemangioblastoma.	Right hemisphere	Depression	Nausea, vomiting, motor deficits	N.S.	N.S.
[105]	55	F	Glioblastoma	Left fronto-parietal	Agoraphobia with panic attacks, depression	Cognitive deficits, sleep-wake disturbances,, speech impediments, motor deficits	3 months	✓
[106]	62	F	Meningioma	Left frontal	Depression	Headache, vomiting, seizure	23 years	✓
[107]	52	F	Glioblastoma	Left thalamus	Personality changes, Psychosis	Cognitive deficits, speech impediments, motor deficits, ocular impairments	1 week	N.S.
[107]	40	F	Meningioma	Lateral ventricle	Personality changes, psychosis, mania	Headache, sleep-wake disturbances, speech impediments, motor deficits	10 months	✓
[107]	32	M	Meningioma	Occipital lobe	Psychosis	Headache, ocular impairments	17 years	✓
[108]	61	F	Meningioma	Frontal	Depression, psychosis	Sleep-wake disturbances, headache, Cognitive deficits, urinary incontinence, motor deficits	3 months	✓
[109]	44	F	Grade III astrocytoma	Frontal, temporal and parietal	Personality changes	Motor deficits, speech impediments	1 year	N.S.
[110]	41	M	Glioblastoma	Left temporal	Depression, anxiety	Headache, sleep-wake disturbances, speech impediments, ocular impairments	7 months	✓
[111]	28	F	Craniopharyngioma	Fronto-temporal	Anorexia nervosa, Apathy	Ocular impairments	4 years	N.S.
[112]	60	F	Meningioma	Frontal	Depression, psychosis	Headache, seizure	2 weeks	✓
[112]	55	M	Grade IV astrocytoma	Right frontal	Depression, psychosis	Headache, cognitive deficits	2 months	✓
[113]	19	F	Germinoma	Third Ventricle	Anorexia nervosa	Dizziness, vomiting, sleep-wake disturbances	5 years	N.S.

[114]	63	F	Meningioma	Left ventricle	Psychosis	Speech impediments	6 months	N.S.
[115]	23	M	Chraniopharyngioma	Suprasellar	Mania	Cognitive deficits, seizure, motor deficits	2 months	N.S.
[116]	36	F	Glioblastoma	Occipital lobe	Psychosis	Cognitive deficits, motor deficits, ocular impairments	N.S.	N.S.
[116]	44	M	Ganglioglioma	Frontal lobe	Psychosis, personality changes	Dizziness, headache, nausea, ocular impairments	4 years	N.S.
[116]	33	F	Meningioma	Frontoparietal	Anxiety	Headache, nausea, seizure, motor deficits	3 years	✓
[116]	50	F	Glioblastoma	Frontoparietal	Personality changes	Dizziness, motor deficits, urinary incontinence, seizure	2 months	N.S.
[116]	60	F	Hemangioblastoma	Fourth Ventricle	Psychosis	Headache, cognitive deficits, motor deficits, ocular impairments	18 months	N.S.
[116]	52	M	Glioblastoma	Fronto-temporal	Psychosis	Headache, cognitive deficits, motor deficits, ocular impairments	unknown	N.S.
[117]	25	F	Astrocytoma	Hypothalamus	Anorexia nervosa	Urinary incontinence, seizure	6 months	N.S.

M: Male, F: Female, N.S.: Not specified, Ref: Reference; ✓: Psychiatric symptoms resolved or improved after tumor resection/treatment, X: Psychiatric symptoms remained after tumor resection/treatment.

Table S3. Characteristics of "Older Adults group" case reports (age ≥ 65 years) with initial psychiatric symptoms with or without generalized and/or neurological signs and symptoms.

Ref.	Age	Gender	Tumor type	Tumor location	Psychiatric symptoms (P.S)	Neurological symptoms	Time from symptoms to diagnosis	P.S after tumor treatment
[118]	72	F	Meningioma	Frontal	Psychosis	Cognitive deficits	9 months	✓
[119]	65	F	Meningioma	N.S.	Apathy, anxiety	Headache	6 months	✓
[120]	68	F	Meningioma	Interhemispheric fissure	Psychosis, personality changes, apathy	Headache, cognitive deficits	3 months	✓
[121]	66	F	Meningioma	Subfrontal	Apathy, personality changes	Headache, urinary incontinence, motor deficits	10 months	N.S.
[122]	68	M	Meningioma	Left parietal	Psychosis	Sleep-wake disturbances, urinary incontinence	10 days	N.S.
[70]	65	M	Meningioma	Suprasellar	Apathy, Personality changes	Cognitive deficits, ocular impairments, motor deficits	6 months	✓
[123]	65	M	Glioblastoma	Parietal, temporal	Apathy	Cognitive deficits, sleep-wake disturbances	4 weeks	N.S.
[124]	68	F	Fibrous Meningioma	Frontal temporal	Depression	Cognitive deficits, sleep-wake disturbances	4 months	✓
[75]	71	F	Grade II Meningioma	Right temporal	Anxiety	Headache, dizziness, cognitive deficits, motor deficits	6 months	N.S.
[125]	79	F	High grade glial neoplasm	Left parietal	Depression, personality changes	Cognitive deficits, sleep-wake disturbances	5 months	✓
[126]	67	F	Astrocytoma	Bilateral thalamic	Apathy	Cognitive deficits	2 months	N.S.
[127]	76	M	Fibroblastic meningio-	Fourth Ventricle	Personality changes	Headache, cognitive deficits, motor defi-	2 weeks	N.S.

			ma			cits		
[93]	66	F	Grade III-IV astrocytoma	Temporoparietal	Anxiety, psychosis	Dizziness, headache, cognitive deficits, seizure, ocular impairments	10 years	N.S.
[128]	66	F	Hemangioma	Temporobasal	Anxiety	Dizziness	3 months	N.S.
[129]	74	F	Meningioma	Left frontal	Apathy	Cognitive deficits, motor deficits, speech impediments	6 months	✓
[129]	76	F	Anaplastic astrocytoma	Left orbitofrontal and right thalamic	Personality changes	Cognitive deficits, motor deficits	N.S.	N.S.
[130]	86	M	Meningioma	Posterior parasagittal	Miscellaneous (Bonnet syndrome)	Motor deficits, ocular impairments	20 years	N.S.
[131]	74	F	Meningioma	Right frontal	Depression	Cognitive deficits, motor deficits, speech impediments, urinary incontinence	2.5 years	N.S.
[132]	75	F	Meningioma	Left frontoparietal	Depression, psychosis	Sleep-wake disturbances	6 months	N.S.
[133]	69	F	Meningioma	Right temporal	Anxiety, depression, psychosis	Cognitive deficits, seizure, speech impediments	3 years	✓
[104]	65	M	Glioblastoma	Left cerebellar	Anxiety, apathy	Dizziness, nausea, vomiting, motor deficits	N.S.	N.S.
[104]	65	M	Astrocytoma	Right cerebellar	Depression	Headache, nausea, motor deficits	2 months	N.S.
[115]	69	M	Glioblastoma	Right Frontal	Personality changes	Headache, cognitive deficits, motor deficits	6 weeks	N.S.
[116]	67	M	Glioblastoma	Left Temporal	Personality changes, psychosis	Dizziness, cognitive deficits	1 year	N.S.

M: Male; F: Female, N.S.: Not specified, Ref: Reference, ✓: Psychiatric symptoms resolved or improved after tumor resection/treatment, X: Psychiatric symptoms remained after tumor resection/treatment.

Table S4. Frequencies of psychiatric or neurological symptoms occurring before the diagnosis of brain tumor in the retrieved case reports according to age groups.

	All cases (n=165)	Pediatric cases (age <18 years, n=33)	Adult cases (18 ≤ age <65 years, n=108)	Older adult cases (age ≥65 years, n=24)	χ^2	p
Psychiatric symptoms						
Anxiety	35 (21.2%)	8 (24.2%)	21 (19.4%)	6 (29.2%)	0.60	0.75
Apathy	25 (15.2%)	1 (3.0%)	16 (14.8 %)	8 (33.3%)	**9.95**	**0.007**
Depression	49 (29.7%)	9 (27.3%)	34 (31.5%)	6 (25.0%)	0.51	0.77
Eating disorder	27 (16.4%)	16 (48.5%)	11 (10.3%)	0 (0%)	**32.59**	**<0.0001**
Manic symptoms	10 (6.1%)	2 (6.1%)	8 (7.4%)	0 (0%)	1.89	0.39
Miscellaneous symptoms	17 (10.3%)	5 (15.2%)	10 (9.3%)	2 (8.3%)	1.07	0.59
Personality changes	37 (22.4%)	5 (15.2%)	24 (22.4%)	8 (33.3%)	2.65	0.27
Psychotic symptoms	62 (37.6%)	10 (30.3%)	45 (41.7%)	7 (25.0%)	2.24	0.33
Neurological symptoms						
Cognitive deficits	65 (39.4%)	8 (24.2%)	41 (38.0%)	16 (66.7%)	**10.74**	**0.005**
Delayed puberty	2 (1.2%)	2 (6.1%)	-	-	**-**	**-**
Dizziness	20 (12.1%)	3 (9.1%)	12 (11.1%)	5 (20.8%)	2.10	0.35
Growth retardation	5 (3.0%)	5 (15.2%)	-	-	**-**	**-**
Headache	59 (35.8%)	9 (27.3%)	42 (38.9%)	8 (33.3%)	1.56	0.46
Motor deficits	66 (40.0%)	17 (51.5%)	38 (35.2%)	11 (45.8%)	3.21	0.20
Nausea/Vomiting	37 (22.4%)	16 (48.5%)	19 (17.6%)	2 (8.3%)	**17.07**	**0.0002**
Ocular impairments	34 (20.6%)	9 (27.3%)	22 (20.4%)	3 (12.5%)	1.86	0.39
Seizures	22 (13.3%)	2 (6.1%)	18 (16.7%)	3 (8.3%)	3.07	0.22
Sleep disturbances	46 (27.9%)	9 (27.3 %)	32 (29.6%)	5 (20.8%)	0.76	0.68
Speech impediments	23 (13.9%)	2 (6.1%)	18 (16.7%)	3 (12.5%)	2.42	0.30
Urinary incontinence	12 (7.3%)	0 (0 %)	9 (8.4%)	3 (12.5%)	3.75	0.15

Significant results are reported in bold.

Table S5. Frequencies of psychiatric or neurological symptoms according to tumor location.

	**Pediatric (age <18 years)**				**Adult (18 ≤ age <65 years)**				Older adult (age ≥65 years)			
	**Supratentorial tumors (n=21)**	**Infratentorial tumors (n=9)**	χ^2	**p**	**Supratentorial tumors (n=91)**	**Infratentorial tumors (n=17)**	χ^2	**p**	**Supratentorial tumors (n=20)**	**Infratentorial tumors (n=3)**	χ^2	**p**
Psychiatric symptoms												
Anxiety	4 (19.1)%	3 (33.3%)	0.72	0.64	17 (18.7%)	4 (23.5%)	0.21	0.74	4 (20.0%)	1 (33.3%)	0.27	10.54
Apathy	0 (0%)	0 (0%)	-	-	15 (16.5%)	1 (5.9%)	1.28	0.46	6 (30.0%)	1 (33.3%)	0.01	1.00
Depression	7 (33.3%)	1 (11.1%)	1.59	0.37	31 (34.1)%	3 (17.6%)	1.79	0.26	5 (25.0%=	1 (33.3%)	0.09	1.00
Eating	10	4 (44.4%)	0.03	1.00	7 (7.7%)	4 (23.5%)	3.93	0.07	0 (0%)	0 (0%)	-	-

disorder	(47.6)%											
Manic symptoms	1 (4.8%)	0 (0%)	0.44	1.00	8 (8.8%)	0 (0%)	1.61	0.35	0 (0%)	0 (0%)	-	-
Miscellaneous	2 (9.5%)	3 (33.3%)	2.57	0.14	6 (6.6%)	4 (23.5%)	**4.89**	**0.049**	2 (10.0)%	0 (0%)	0.33	1.00
Personality changes	4 (19.1)%	1 (11.1)%	0.29	1.00	22 (24.2%)	2 (11.8%)	1.28	0.35	7 (35.0%)	1 (33.3%)	0.00	1.00
Psychotic symptoms	8 (38.1%)	1 (11.1)%	2.18	0.21	39 (42.9%)	6 (35.3%)	0.34	0.60	7 (35.0%)	0 (0%)	1.51	0.53
Neurological symptoms												
Cognitive deficits	6 (28.6%)	1 (11.1)%	1.07	0.30	36 (39.6%)	5 (29.4%)	0.63	0.59	15 (74%)	1 (33.3%)	2.14	0.21
Delayed puberty	2 (9.5%)	0 (0%)	0.92	1.00	0 (0%)	0 (0%)	-	-	0 (0%)	0 (0%)	-	-
Dizziness	2 (9.5%)	1 (11.1%)	0.02	1.00	8 (8.8%)	4 (23.5%)	3.15	0.09	4 (20.0%)	1 (33.3%)	0.27	0.54
Growth retardation	4 (19.1%)	0 (0%)	1.98	0.29	0 (0%)	0 (0%)	-	-	0 (0%)	0 (0%)	-	-
Headache	9 (42.9%)	0 (0%)	**5.51**	**0.029**	34 (37.4%)	8 (47.1%)	0.57	0.59	5 (25.0%)	2 (66.7%)	2.14	0.21
Motor deficits	10 (47.6%)	6 (66.7%)	0.92	0.44	29 (31.9%)	9 (52.9%)	2.79	0.11	8 (40.0%)	3 (100%)	3.77	0.09
Nausea/Vomiting	10 (47.6%)	4 (44.4%)	0.03	1.00	15 (16.5%)	4 (23.5%)	0.49	0.50	0 (0%)	2 (66.7%)	**14.60**	**0.012**
Ocular impairments	9 (42.9%)	0 (0%)	**5.51**	**0.029**	19 (20.9%)	3 (17.6%)	0.09	1.00	3 (15.0%)	0 (0%)	0.52	1.00
Seizures	1 (4.8%)	1 (11.1%)	0.41	0.52	17 (18.7%)	1 (5.9%)	1.69	0.30	2 (10.0%)	0 (0%)	0.33	1.00
cSleep disturbances	6 (28.6%)	22%	0.13	1.00	31 (34.1%)	1 (5.9%)	**5.46**	**0.02**	5 (25.0%)	0 (0%)	0.96	1.00
Speech impediments	0 (0%)	0 (0%)	-	-	16 (17.6%)	2 (11.8%)	0.35	0.73	3 (15.0%)	0 (0%)	0.52	1.00
Urinary incontinence	0 (0%)	0 (0%)	-	-	7 (7.7%)	2 (11.8%)	0.30	0.63	3 (15.0%)	0 (0%)	0.52	1.00

Brain tumors were classified in supratentorial or infratemporal tumors based on the reported tumor location. Two pediatric cases with both supra and infratemporal location were excluded from the analyses. Significant results are reported in bold.

Table S6. Frequencies of the case reports with initial psychiatric symptoms with or without generalized and/or neurological signs and symptoms, according to age groups.

	Cases that recorded psychiatric symptoms only	Cases that recorded psychiatric & generalized neurological symptoms only	When present, presenting generalized symptoms appeared		When present,focal neurological symptoms appeared		Average duration of delay until tumor diagnosis (months)	Recorded cases whose psychiatric symptoms after tumor resection/ treatment	
			Along with psychiatric symptoms	After the incidence of psychiatric symptoms	Along with psychiatric symptoms	After the incidence of psychiatric symptoms		Resolved or Improved	Remained
**All cases (n=165)**	**17 (10.3%)**	**63 (38.2%)**	75 (58.1%)	54 (41.8%)	26 (25.5%)	76 (74.5%)	31± 53.3	93 (93%)	7 (7%)
			Total = 129 (78.2%)		Total = 102 (61.8%)			Total = 100	
Age <18 years (n=33)	2(6.1%)	8 (24.2%)	18 (66.7%)	9 (33.3%)	4 (17.4%)	19 (82.6%)	17.9 ± 20.6	23 (92%)	2 (8%)
			Total = 27 (81.8%)		Total = 23 (69.7%)			Total = 25	
18≤ age <65 years (n=108)	15 (13.9%)	43 (39.8%)	39 (49.4%)	40(50.6%)	16 (24.6%)	49 (75.4%)	37± 59.3	62 (92.5%)	5 (7.5%)
			Total = 79 (73.1%)		Total = 65 (60.2%)			Total = 67	
Age ≥ 65 years (n=24)	0(0%)	10 (41.7%)	17 (73.9%)	6 (26.1%)	6 (42.9%)	8 (57.1%)	23.2 ± 54.8	8 (100%)	0
			Total = 23 (95.8%)		Total = 14 (58.3%)			Total = 8	

Average duration of delay until tumor diagnosis is expressed as Mean ± S.D. (in months).

List of references of supplementary tables:

1. Hamamoto Filho, P.T.; Magro, I.B.; Zanini, M.A.; Hamamoto Filho, P.T.; Magro, I.B.; Zanini, M.A. Behavioural Changes Caused by Diffuse Intrinsic Pontine Glioma. *Revista da Associação Médica Brasileira* **2018**, *64*, 581–582, doi:10.1590/1806-9282.64.07.581.

2. Yuge, K.; Ohya, T.; Shibuya, I.; Nagamitsu, S.; Yamashita, Y. Pathological Crying and Emotional Vasovagal Syncope as Symptoms of a Dorsally Exophytic Medullary Tumor. *Brain Dev* **2016**, *38*, 609–12, doi:10.1016/j.braindev.2015.12.008.
3. Krayem, B.H.; Dunn, N.R.; Swift, R.G. Psychosis after Right Temporal Lobe Tumor Resection and Recurrence. *J Neuropsychiatry Clin Neurosci* **2014**, *26*, E47, doi:10.1176/appi.neuropsych.13030051.
4. Hensgens, T.B.; Bloemer, E.; Schouten-van Meeteren, A.Y.N.; Zwaan, C.M.; Van den Bos, C.; Huyser, C.; Kaspers, G.J.L. Psychiatric Symptoms Causing Delay in Diagnosing Childhood Cancer: Two Case Reports and Literature Review. *Eur Child Adolesc Psychiatry* **2013**, *22*, 443–450, doi:10.1007/s00787-012-0349-7.
5. Kaloshi, G.; Alikaj, V.; Rroji, A.; Vreto, G.; Petrela, M. Visual and Auditory Hallucinations Revealing Cerebellar Extraventricular Neurocytoma: Uncommon Presentation for Uncommon Tumor in Uncommon Location. *Gen Hosp Psychiatry* **2013**, *35*, 680.e1–3, doi:10.1016/j.genhosppsych.2013.03.011.
6. Webb, T.N.; Patel, S.M.; Chase, A.; Sankararaman, S.; Patra, K.P.; Gupta, R.; Jeroudi, M. Index of Suspicion. Case 1: Fever, Dysuria, and Abdominal Pain and Distension in a 3-Year-Old Girl. Case 2: Behavioral Changes and Staring Spells in a Healthy 8-Year-Old Boy. Case 3: Intermittent Headaches, Calf Pain, and Fatigue in a 6-Year-Old Boy. *Pediatr Rev* **2013**, *34*, 235–241, doi:10.1542/pir.34-5-235.
7. Undurraga, J.; Baeza, I.; Valentí, M.; Lázaro, M.L. Brain Germinoma Presenting as a First Psychotic Episode in an Adolescent Male. *Eur Child Adolesc Psychiatry* **2010**, *19*, 741–742, doi:10.1007/s00787-010-0107-7.
8. Mittal, V.A.; Karlsgodt, K.; Zinberg, J.; Cannon, T.D.; Bearden, C.E. Identification and Treatment of a Pineal Region Tumor in an Adolescent with Prodromal Psychotic Symptoms. *Am J Psychiatry* **2010**, *167*, 1033–1037, doi:10.1176/appi.ajp.2010.09071043.
9. Tamburin, S.; Cacciatori, C.; Bonato, C.; Zanette, G. Cingulate Gyrus Tumor Presenting as Panic Attacks. *Am J Psychiatry* **2008**, *165*, 651–652, doi:10.1176/appi.ajp.2007.07061005.
10. Oreskovic, N.M.; Strother, C.G.; Zibners, L.M. An Unusual Case of a Central Nervous System Tumor Presenting as a Chief Complaint of Depression. *Pediatr Emerg Care* **2007**, *23*, 486–488, doi:10.1097/01.pec.0000280522.22946.a7.
11. Rohrer, T.R.; Fahlbusch, R.; Buchfelder, M.; Dörr, H.G. Craniopharyngioma in a Female Adolescent Presenting with Symptoms of Anorexia Nervosa. *Klin Padiatr* **2006**, *218*, 67–71, doi:10.1055/s-2006-921506.

12. Distelmaier, F.; Janssen, G.; Mayatepek, E.; Schaper, J.; Göbel, U.; Rosenbaum, T. Disseminated Pilocytic Astrocytoma Involving Brain Stem and Diencephalon: A History of Atypical Eating Disorder and Diagnostic Delay. *J. Neurooncol.* **2006**, *79*, 197–201, doi:10.1007/s11060-006-9125-1.
13. Hargrave, D.R.; Mabbott, D.J.; Bouffet, E. Pathological Laughter and Behavioural Change in Childhood Pontine Glioma. *J. Neurooncol.* **2006**, *77*, 267–271, doi:10.1007/s11060-005-9034-8.
14. Velázquez Fragua, R.; Méndez Echevarría, A.; Cazorla Calleja, M.R.; Baquero-Artigao, F.; Jordán Jiménez, A. [Pinealoma presenting as a psychiatric disorder]. *An Pediatr (Barc)* **2004**, *61*, 448–449, doi:10.1016/s1695-4033(04)78426-x.
15. Bos, R.F.; Ramaker, C.; van Ouwerkerk, W.J.R.; Linssen, W.H.J.P.; Wolf, B.H.M. [Vomiting as a first neurological sign of brain tumors in children]. *Ned Tijdschr Geneeskd* **2002**, *146*, 1393–1398.
16. Lehrnbecher, T.; Kellner, M.; Warmuth-Metz, M.; Kühl, J.; Müller, H.-L. Fehldiagnose Anorexia nervosa bei 2 Patienten mit Malignom. *Monatsschr Kinderheilkd* **2001**, *149*, 914–917, doi:10.1007/s001120170085.
17. Craven, C. Pineal Germinoma and Psychosis. *J Am Acad Child Adolesc Psychiatry* **2001**, *40*, 6, doi:10.1097/00004583-200101000-00008.
18. O'Brien, A.; Hugo, P.; Stapleton, S.; Lask, B. "Anorexia Saved My Life": Coincidental Anorexia Nervosa and Cerebral Meningioma. *Int J Eat Disord* **2001**, *30*, 346–349.
19. Mordecai, D.; Shaw, R.J.; Fisher, P.G.; Mittelstadt, P.A.; Guterman, T.; Donaldson, S.S. Case Study: Suprasellar Germinoma Presenting With Psychotic and Obsessive-Compulsive Symptoms. *Journal of the American Academy of Child & Adolescent Psychiatry* **2000**, *39*, 116–119, doi:10.1097/00004583-200001000-00024.
20. Nadvi, S.S.; Ramdial, P.K. Transient Peduncular Hallucinations Secondary to Brain Stem Compression by a Cerebellar Pilocytic Astrocytoma. *Br J Neurosurg* **1998**, *12*, 579–581.
21. Carson, B.S.; Weingart, J.D.; Guarnieri, M.; Fisher, P.G. Third Ventricular Choroid Plexus Papilloma with Psychosis: Case Report. *Journal of Neurosurgery* **1997**, *87*, 103–105, doi:10.3171/jns.1997.87.1.0103.
22. De Vile, C.J.; Sufraz, R.; Lask, B.D.; Stanhope, R. Occult Intracranial Tumours Masquerading as Early Onset Anorexia Nervosa. *BMJ* **1995**, *311*, 1359–1360.
23. Nadvi, S.S.; van Dellen, J.R. Transient Peduncular Hallucinations Secondary to Brain Stem Compression by a Medulloblastoma. *Surgical Neurology* **1994**, *41*, 250–252, doi:10.1016/0090-3019(94)90132-5.
24. Chipkevitch, E.; Fernandes, A.C. Hypothalamic Tumor Associated with Atypical Forms of Anorexia Nervosa and Diencephalic Syndrome. *Arq Neuropsiquiatr* **1993**, *51*, 270–274.
25. Berek, K.; Aichner, F.; Schmutzhard, E.; Kofler, M.; Langmayr, J.; Gerstenbrand, F. Intracranial Germ Cell Tumor Mimicking Anorexia Nervosa. *Klin Wochenschr* **1991**, *69*, 440–442, doi:10.1007/BF01666831.
26. Wright, K.; Smith, M.S.; Mitchell, J. Organic Diseases Mimicking Atypical Eating Disorders. *Clin Pediatr (Phila)* **1990**, *29*, 325–328, doi:10.1177/000992289002900606.
27. Blackman, M.; Wheler, G.H. A Case of Mistaken Identity: A Fourth Ventricular Tumor Presenting as School Phobia in a 12 Year Old Boy. *Can J Psychiatry* **1987**, *32*, 584–587.
28. Biebl, W.; Platz, T.; Kinzl, J.; Aichner, F. [A case of atypical male anorexia nervosa: a tumor in the area of the 3d ventricle]. *Nervenarzt* **1984**, *55*, 265–268.
29. Maroon, J.C.; Albright, L. "Failure to Thrive" Due to Pontine Glioma. *Arch. Neurol.* **1977**, *34*, 295–297.
30. Burr, I.M.; Slonim, A.E.; Danish, R.K.; Gadoth, N.; Butler, I.J. Diencephalic Syndrome Revisited. *J. Pediatr.* **1976**, *88*, 439–444.
31. Trevizol, A.P.; Cerqueira, R. de O.; Brietzke, E.; Cordeiro, Q.; Trevizol, A.P.; Cerqueira, R. de O.; Brietzke, E.; Cordeiro, Q. New-Onset Psychiatric Symptoms Following Intracranial Meningioma in a Patient with Schizophrenia: A Case Study. *Brazilian Journal of Psychiatry* **2019**, *41*, 91–92, doi:10.1590/1516-4446-2018-0055.
32. Pal, A.; Gondwal, R.; Saxena, V.; Avinash, P.R. Godot Syndrome: A Rare Presentation of Anxiety in a Young Male with Glioblastoma Multiforme. *Asian J Psychiatr* **2019**, *40*, 68–70, doi:10.1016/j.ajp.2019.01.013.
33. Antunes, C.; Ramos, R.; Machado, M.J.; Filipe, M.A. Giant Posterior Fossa Meningioma: The Importance of Early Diagnosis and Challenges Concerning Treatment. *BMJ Case Rep* **2019**, *12*, doi:10.1136/bcr-2018-228454.
34. Gosal, J.S.; Pandey, S.; Das, K.K.; Khatri, D.; Rangari, K.; Jaiswal, A.K.; Behari, S. Pathologic Laughter as an Early and Unusual Presenting Symptom of Petroclival Meningioma: A Case Report and Review of the Literature. *World Neurosurg* **2019**, *123*, 161–164, doi:10.1016/j.wneu.2018.11.240.
35. Schildermans, J. [Psychosis and behavioural problems as first symptoms of brain tumour?]. *Tijdschr Psychiatr* **2019**, *61*, 53–56.

36. Hussin, S.; Yusoff, S.S.M.; Zin, F.M.; Ghani, A.R.I. Frontal Meningioma with Psychiatric Symptoms. *J Family Med Prim Care* **2018**, *7*, 252–254, doi:10.4103/jfmpc.jfmpc_157_17.
37. Akan, Mustafa; Erbay, Lale Goneni; Erbay, Mehmet Fatih; Unal, Suheyla Brain Tumors and Atypical Psychiatric Symptoms: Two Case Presentations. *Dusunen Adam: Journal of Psychiatry & Neurological Sciences* **Sep2018**, *31*, 326–328.
38. Estronza, S.; Saavedra, F.M.; De Jesus, O.; Pastrana, E.A. Chordoid Glioma with Psychosis: Case Report. *P R Health Sci J* **2018**, *37*, 174–176.
39. Petzold, J.; Severus, E.; Meyer, S.; Bauer, M.; Daubner, D.; Krex, D.; Juratli, T.A. Glioblastoma Multiforme Presenting as Postpartum Depression: A Case Report. *J Med Case Rep* **2018**, *12*, 374, doi:10.1186/s13256-018-1909-3.
40. Karakula-Juchnowicz, H.; Morylowska-Topolska, J.; Juchnowicz, D.; Korzeniowska, A.; Krukow, P.; Rola, R. Paranoid Syndrome as the First Sign of Central Neurocytoma: A Case Report. *J Psychiatr Pract* **2018**, *24*, 359–363, doi:10.1097/PRA.0000000000000332.
41. Dutschke, L.L.; Steinau, S.; Wiest, R.; Walther, S. Brain Tumor-Associated Psychosis and Spirituality—A Case Report. *Front Psychiatry* **2017**, *8*, doi:10.3389/fpsyt.2017.00237.
42. Takeuchi, N.; Kato, E.; Kanemoto, K. Obsessive-Compulsive Disorder Associated with Posterior Cranial Fossa Meningioma Available online: https://www.hindawi.com/journals/crips/2017/8164537/ (accessed on 20 November 2018).
43. Bilanakis, N.; Vratsista, A.; Siorou, S. A Brain Ependymoma with Psychiatric Manifestation. *J BUON* **2017**, *22*, 1604–1605.
44. Singh, P.; Khan, A.; Scott, G.; Jasper, M.; Singh, E. Lesson of the Month 2: A Choroid Plexus Papilloma Manifesting as Anorexia Nervosa in an Adult. *Clin Med (Lond)* **2017**, *17*, 183–185, doi:10.7861/clinmedicine.17-2-183.
45. Chaari, I.; Ben Ammar, H.; Nefzi, R.; Mhedhbi, N.; Khelifa, E.; Aissa, A.; El Hechmi, Z. Frontal Meningioma and Bipolar Disorder: Etiopathogenic Link or Co-Morbidity? A Case Report. *European Psychiatry* **2017**, *41*, S678, doi:10.1016/j.eurpsy.2017.01.1170.
46. Shukla, A.; Das, A.; Behere, P. Psychiatric Symptoms in Brain Tumor. *Eastern Journal of Psychiatry* **2017**, *18*.
47. Ceylan, E.M.; Önen Ünsalver, B.; Evrensel, A. Medial Cranial Fossa Meningioma Diagnosed as Mixed Anxiety Disorder with Dissociative Symptoms and Vertigo Available online: https://www.hindawi.com/journals/crips/2016/3827547/ (accessed on 20 November 2018).
48. Munjal, S.; Pahlajani, S.; Baxi, A.; Ferrando, S. Delayed Diagnosis of Glioblastoma Multiforme Presenting With Atypical Psychiatric Symptoms. *Prim Care Companion CNS Disord* **2016**, *18*, doi:10.4088/PCC.16l01972.
49. Yapici-Eser, H.; Onay, A.; Oztop-Cakmak, O.; Egemen, E.; Vanli-Yavuz, E.N.; Solaroglu, I. Rare Case of Glioblastoma Multiforme Located in Posterior Corpus Callosum Presenting with Depressive Symptoms and Visual Memory Deficits. *BMJ Case Rep* **2016**, *2016*, doi:10.1136/bcr-2016-216505.
50. Yakhmi, S.; Sidhu, B.S.; Kaur, J.; Kaur, A. Diagnosis of Frontal Meningioma Presenting with Psychiatric Symptoms. *Indian J Psychiatry* **2015**, *57*, 91–93, doi:10.4103/0019-5545.148534.
51. Dautricourt, S.; Marzloff, V.; Dollfus, S. Meningiomatosis Revealed by a Major Depressive Syndrome. *BMJ Case Rep* **2015**, *2015*, doi:10.1136/bcr-2015-211909.
52. Saha, R.; Jakhar, K. Oligodendroglioma Presenting as Chronic Mania. *Shanghai Arch Psychiatry* **2015**, *27*, 183–185, doi:10.11919/j.issn.1002-0829.215039.
53. Chen, H.-C.; Lin, C.-F.; Lee, Y.-C. The Right Amygdalar Tumor Presenting with Symptoms of Separation Anxiety Disorder (SAD): A Case Report. *Neurocase* **2015**, *21*, 268–270, doi:10.1080/13554794.2014.892620.
54. Hussain, T.; Shafat, M.; Ahmad Bhat, J. Frontal Lobe Meningioma Masquerading as Depressive Disorder. *Journal of Psychiatry* **2015**, *18*, doi:10.4172/2378-5756.1000328.
55. Zugman, A.; Pan, P.M.; Gadelha, A.; Mansur, R.B.; Asevedo, E.; Cunha, G.R.; Silva, P.F.R.; Brietzke, E.; Bressan, R.A. Brain Tumor in a Patient with Attenuated Psychosis Syndrome. *Schizophr. Res.* **2013**, *144*, 151–152, doi:10.1016/j.schres.2012.11.036.
56. Mumoli, N.; Pulerà, F.; Vitale, J.; Camaiti, A. Frontal Lobe Syndrome Caused by a Giant Meningioma Presenting as Depression and Bipolar Disorder. *Singapore Med J* **2013**, *54*, e158-159.
57. Arasappa, R.; Danivas, V.; Venkatasubramanian, G. Choroid Plexus Papilloma Presenting as Schizophrenia: A Case Report. *J Neuropsychiatry Clin Neurosci* **2013**, *25*, E26-27, doi:10.1176/appi.neuropsych.12010017.
58. Abecassis, I.J.; Smith, T.; Chandler, J.P. Brain Tumors and the Area Postrema. *J Clin Neurosci* **2013**, *20*, 1795–1797, doi:10.1016/j.jocn.2013.01.028.

59. Pawelczyk, A.; Lojek, E.; Rabe-Jablonska, J.; Pawelczyk, T.; Godlewski, B.; Radek, M. Depression or Apathy? A Diagnostic Trap: A Huge Right Frontal Lobe Meningioma Diagnosed and Treated as Mild Atypical Depression Episode-A Case Study. *Psychiatria Polska* **2012**, *46*, 903–913.
60. Goddard, E.; Ashkan, K.; Farrimond, S.; Bunnage, M.; Treasure, J. Right Frontal Lobe Glioma Presenting as Anorexia Nervosa: Further Evidence Implicating Dorsal Anterior Cingulate as an Area of Dysfunction. *Int J Eat Disord* **2013**, *46*, 189–192, doi:10.1002/eat.22072.
61. Assefa, D.; Haque, F.N.; Wong, A.H. Case Report: Anxiety and Fear in a Patient with Meningioma Compressing the Left Amygdala. *Neurocase* **2012**, *18*, 91–94, doi:10.1080/13554794.2011.556126.
62. Mirsattari, S.M.; Gofton, T.E.; Chong, D.J. Misdiagnosis of Epileptic Seizures as Manifestations of Psychiatric Illnesses. *Can J Neurol Sci* **2011**, *38*, 487–493.
63. Mignogna, M.D.; Adamo, D.; Falleti, J.; Fortuna, G. Dysgeusia: An Atypical and Neglected Psychiatric Symptom Induced by Fibrillary Astrocytoma. *Am. J. Med.* **2011**, *124*, e1-2, doi:10.1016/j.amjmed.2011.01.008.
64. Betul, O.; Ipek, M. Brain Tumor Presenting With Psychiatric Symptoms. *JNP* **2011**, *23*, E43–E44, doi:10.1176/jnp.23.4.jnpe43.
65. Canuet, L.; Ikezawa, K.; Ishii, R.; Aoki, Y.; Iwase, M.; Takeda, M. Schizophrenia-like Psychosis Associated with Right-Parietal Meningioma. *J Neuropsychiatry Clin Neurosci* **2011**, *23*, E36, doi:10.1176/jnp.23.3.jnpe36.
66. Sarkheil, P.; Werner, C.J.; Mull, M.; Schneider, F.; Neuner, I. Depressive Episode Induced by Frontal Tumor Culminating in Suicidal Ideation.; 2010.
67. Cheema, F.A.; Badr, A.; Iqbal, J. Glioblastoma Multiforme Presenting as Treatment-Resistant Depression. *J Neuropsychiatry Clin Neurosci* **2010**, *22*, 123.E26, doi:10.1176/jnp.2010.22.1.123.e26.
68. Rosenzweig, I.; Bodi, I.; Selway, R.P.; Crook, W.S.; Moriarty, J.; Elwes, R.D.C. Paroxysmal Ictal Phonemes in a Patient with Angiocentric Glioma. *J Neuropsychiatry Clin Neurosci* **2010**, *22*, 123.E18–20, doi:10.1176/jnp.2010.22.1.123.e18.
69. Tsai, M.-C.; Huang, T.-L. Generalized Anxiety Disorder in a Patient Prior to the Diagnosis of Left Temporal Lobe Meningioma: A Case Report. *Progress in Neuro-Psychopharmacology & Biological Psychiatry* **2009**, *33*, 1082–1083, doi:10.1016/j.pnpbp.2009.05.021.
70. Cretin, B.; Echaniz-Laguna, A.; Meyer, C.; Blanc, F.; Sellal, F. Apathy or Depression? Do You Have a Nose for It? Four Case Reports of Paramedian Frontal Tumors. *Revue Neurologique* **2010**, *166*, 704–710.
71. Vad Winkler, L. Slow-Growing Craniopharyngioma Masquarading as Early-Onset Eating Disorder: Two Cases. *International Journal of Eating Disorders* **2009**, *42*, 475–478, doi:10.1002/eat.20635.
72. Tsutsumi, S.; Yasumoto, Y.; Ito, M. Pathological Laughter Caused by Frontal Glioblastoma: Case Report. *Neurol. Med. Chir. (Tokyo)* **2008**, *48*, 307–310.
73. Ozcan, S.; Evran, M.; Koc, F.; Saatci, E. Glioblastoma Multiforme Presenting With Psychiatric Symptoms in a Primary Care Setting: Review of Isolated Psychiatric Symptoms With Brain Tumors. *Neurosurgery Quarterly* **2008**, *18*, 148, doi:10.1097/WNQ.0b013e318172f980.
74. Habermeyer, B.; Weiland, M.; Mager, R.; Wiesbeck, G.A.; Wurst, F.M. A Clinical Lesson: Glioblastoma Multiforme Masquerading as Depression in a Chronic Alcoholic. *Alcohol Alcohol* **2008**, *43*, 31–3, doi:10.1093/alcalc/agm150.
75. Bunevicius, A.; Deltuva, V.P.; Deltuviene, D.; Tamasauskas, A.; Bunevicius, R. Brain Lesions Manifesting as Psychiatric Disorders: Eight Cases. *CNS Spectr* **2008**, *13*, 950–958.
76. Houy, E.; Debono, B.; Dechelotte, P.; Thibaut, F. Anorexia Nervosa Associated with Right Frontal Brain Lesion. *Int J Eat Disord* **2007**, *40*, 758–761, doi:10.1002/eat.20439.
77. Famularo, G.; Corsi, F.M.; Minisola, G.; De Simone, C.; Nicotra, G.C. Cerebellar Tumour Presenting with Pathological Laughter and Gelastic Syncope. *Eur. J. Neurol.* **2007**, *14*, 940–943, doi:10.1111/j.1468-1331.2007.01784.x.
78. Winston, A.P.; Barnard, D.; D'Souza, G.; Shad, A.; Sherlala, K.; Sidhu, J.; Singh, S.P. Pineal Germinoma Presenting as Anorexia Nervosa: Case Report and Review of the Literature. *Int J Eat Disord* **2006**, *39*, 606–608, doi:10.1002/eat.20322.
79. Moise, D.; Madhusoodanan, S. Psychiatric Symptoms Associated with Brain Tumors: A Clinical Enigma. *CNS Spectr* **2006**, *11*, 28–31.
80. Pavesi, G.; Berlucchi, S.; Feletti, A.; Opocher, G.; Scienza, R. Hemangioblastoma of the Obex Mimicking Anorexia Nervosa. *Neurology* **2006**, *67*, 178–179, doi:10.1212/01.wnl.0000223354.86636.ed.
81. Shiferaw, K.; Pizzolato, G.P.; Perret, G.; Harpe, R.L. Sudden, Unexpected Death Due to Undiagnosed Frontal Glioblastoma in a Schizophrenic Patient. *Forensic Sci. Int.* **2006**, *158*, 200–203, doi:10.1016/j.forsciint.2005.08.006.

82. Baumann, C.R.; Regard, M.; Trier, S.; Schuknecht, B.; Siegel, A.M. Lipoma on the Corpus Callosum in a Patient with Schizophrenia-like Episode: Is There a Causal Relationship? *Cogn Behav Neurol* **2006**, *19*, 109–111, doi:10.1097/01.wnn.0000209871.46651.73.
83. Ouma, J. Psychotic Manifestations in Brain Tumour Patients: 2 Case Reports from South Africa. *Afr Health Sci* **2004**, *4*, 190–194.
84. Sinai, J.; Wong, A.H.C. Craniopharyngeoma Presenting as Psychosis, Disinhibition and Personality Change without Neurological Signs. *Acta Neuropsychiatrica* **2003**, *15*, 94–96, doi:10.1034/j.1601-5215.2003.00013.x.
85. Sokolski, K.N.; Denson, T.F. Exacerbation of Mania Secondary to Right Temporal Lobe Astrocytoma in a Bipolar Patient Previously Stabilized on Valproate. *Cogn Behav Neurol* **2003**, *16*, 234–238.
86. Burns, J.M.; Swerdlow, R.H. Right Orbitofrontal Tumor With Pedophilia Symptom and Constructional Apraxia Sign. *Arch Neurol* **2003**, *60*, 437–440, doi:10.1001/archneur.60.3.437.
87. Mearin-Manrique, I.; Aragües-Figuero, M.; Jiménez-Arriero, M.A.; Palomo, T. [Depressive disorder and craniopharyngioma]. *Rev Neurol* **2003**, *37*, 999–1000.
88. Miyazawa, T.; Fukui, S.; Otani, N.; Tsuzuki, N.; Katoh, H.; Ishihara, S.; Nawashiro, H.; Wada, K.; Shima, K. Peduncular Hallucinosis Due to a Pineal Meningioma. Case Report. *J. Neurosurg.* **2001**, *95*, 500–502, doi:10.3171/jns.2001.95.3.0500.
89. Muzumdar, D.; Agrahar, P.; Desai, K.; Goel, A. Pathological Laughter as a Presenting Symptom of Petroclival Meningioma--Case Report. *Neurol. Med. Chir. (Tokyo)* **2001**, *41*, 505–507, doi:10.2176/nmc.41.505.
90. Tsutsumi, S.; Hatashita, S.; Kadota, Y.; Abe, K.; Ueno, H. Tentorial Meningioma Associated with Pathological Laughter--Case Report. *Neurol Med Chir (Tokyo)* **2000**, *40*, 272–4, doi:10.2176/nmc.40.272.
91. Mazure, C.M.; Leibowitz, K.; Bowers, M.B. Drug-Responsive Mania in a Man with a Brain Tumor. *J Neuropsychiatry Clin Neurosci* **1999**, *11*, 114–115, doi:10.1176/jnp.11.1.114.
92. Shafqat, S.; Elkind, M.S.; Chiocca, E.A.; Takeoka, M.; Koroshetz, W.J. Petroclival Meningioma Presenting with Pathological Laughter. *Neurology* **1998**, *50*, 1918–1919, doi:10.1212/wnl.50.6.1918.
93. Lilja, A.; Salford, L.G. Early Mental Changes in Patients with Astrocytomas with Special Reference to Anxiety and Epilepsy. *PSP* **1997**, *30*, 316–323, doi:10.1159/000285074.
94. John, G.; Eapen, V.; Shaw, G.K. Frontal Glioma Presenting as Anxiety and Obsessions: A Case Report. *Acta Neurol. Scand.* **1997**, *96*, 194–195, doi:10.1111/j.1600-0404.1997.tb00266.x.
95. Ball, C. The Psychiatric Presentation of a Cerebellopontine Angle Tumour. *Irish Journal of Psychological Medicine* **1996**, *13*, 21–23, doi:10.1017/S079096670000224X.
96. Spence, S.A.; Taylor, D.G.; Hirsch, S.R. Depressive Disorder Due to Craniopharyngioma. *J R Soc Med* **1995**, *88*, 637–638.
97. Filley, C.M.; Kleinschmidt-DeMasters, B.K. Neurobehavioral Presentations of Brain Neoplasms. *West. J. Med.* **1995**, *163*, 19–25.
98. Fahy, S.T.; Carey, T.G.; Owens, J.M.; Owens, A.P. Psychiatric Presentation of Frontal Meningiomas. *Irish Journal of Psychological Medicine* **1995**, *12*, 61–63, doi:10.1017/S0790966700004225.
99. Maurice-Williams, R.S.; Dunwoody, G. Late Diagnosis of Frontal Meningiomas Presenting with Psychiatric Symptoms. *Br Med J (Clin Res Ed)* **1988**, *296*, 1785–1786.
100. Roberts, J.A.; Williams, D.J.; Stack, B.H. Meningioma in a Chronic Schizophrenic. *Scott Med J* **1987**, *32*, 83–84, doi:10.1177/003693308703200311.
101. Summerfield, D.A. Psychiatric Vulnerability and Cerebellar Haemangioblastoma. A Case Report. *Br J Psychiatry* **1987**, *150*, 858–860.
102. Uribe, V.M. Psychiatric Symptoms and Brain Tumor. *Am Fam Physician* **1986**, *34*, 95–98.
103. Dyck, P. Sylvian Lipoma Causing Auditory Hallucinations: Case Report. *Neurosurgery* **1985**, *16*, 64–67, doi:10.1097/00006123-198501000-00013.
104. Rabey, J.M.; Avrahami, E. Unmasking of Cerebellar Tumours by Amitriptyline in Depressive Patients. *J Neurol Neurosurg Psychiatry* **1985**, *48*, 291, doi:10.1136/jnnp.48.3.291.
105. Dietch, J.T. Cerebral Tumor Presenting with Panic Attacks. *Psychosomatics* **1984**, *25*, 861–863, doi:10.1016/S0033-3182(84)72948-3.
106. Maurice-Williams, R.S.; Sinar, E.J. Depression Caused by an Intracranial Meningioma Relieved by Leucotomy Prior to Diagnosis of the Tumour. *J Neurol Neurosurg Psychiatry* **1984**, *47*, 884–885.
107. Binder, R.L. Neurologically Silent Brain Tumors in Psychiatric Hospital Admissions: Three Cases and a Review. *J Clin Psychiatry* **1983**, *44*, 94–97.

108. Lahmeyer, H.W. Frontal Lobe Meningioma and Depression. *J Clin Psychiatry* **1982**, *43*, 254–255.
109. Peterson, L.G.; Perl, M. Psychiatric Presentations of Cancer. *Psychosomatics* **1982**, *23*, 601–4, doi:10.1016/s0033-3182(82)73360-2.
110. Littman, S.T.; Marinchak, P.; Cohen, K.D. A Depressed Patient with Mild Speech Impediment. *Biol. Psychiatry* **1981**, *16*, 589–591.
111. Goldney, R.D. Craniopharyngioma Simulating Anorexia Nervosa. *J. Nerv. Ment. Dis.* **1978**, *166*, 135–138.
112. Carlson, R.J. Frontal Lobe Lesions Masquerading as Psychiatric Disturbances. *Can Psychiatr Assoc J* **1977**, *22*, 315–318.
113. Hollatz, F.; Ziolko, H.U. [The differential diagnosis of anorexia nervosa. Coincidence of somatic disease and psychogenic emaciation (author's transl)]. [German]. *MMW - Munchener Medizinische Wochenschrift* **1976**, *118*, 263–266.
114. Buchanan, D.C.; Abram, H.S. Psychotic Behavior Resulting from a Lateral Ventricle Meningioma: A Case Report. *Dis Nerv Syst* **1975**, *36*, 400–401.
115. Withersty, D.J. Brain Tumors Presenting with Psychiatric Symptomatology. (A Five-Year Survey). *W V Med J* **1974**, *70*, 51–53.
116. Donald, A.G.; Still, C.N.; Pearson, J.M. Behavioral Symptoms with Intracranial Neoplasm. *South. Med. J.* **1972**, *65*, 1006–1009.
117. Lewin, K.; Mattingly, D.; Millis, R.R. Anorexia Nervosa Associated with Hypothalamic Tumour. *Br Med J* **1972**, *2*, 629–630.
118. Byrne, A.; Henry, S. Meningioma and Psychosis – Cause or Coincidence? *Progress in Neurology and Psychiatry* **2020**, *24*, 11–15, doi:https://doi.org/10.1002/pnp.672.
119. Mardaga, S.; Al, M.B.; Bracke, J.; Dutilleux, A.; Born, J.D. [Which psychiatric symptoms must raise suspicion about a possible brain tumor◎?]. *Rev Med Liege* **2017**, *72*, 399–405.
120. Velakoulis, D.; Gleason, A.; Hayhow, B.; McNeil, P.; Gaillard, F. Frontal Meningioma Mimicking Relapse of Schizophrenia. *Aust N Z J Psychiatry* **2014**, *48*, 486–487, doi:10.1177/0004867413511998.
121. Aydin, E.F.; Ozan, E. Transience of Dysexecutive Syndrome but Permanence of Motor Deficits in the Course of Recurrent Subfrontal Meningioma. *J Neuropsychiatry Clin Neurosci* **2013**, *25*, E19, doi:10.1176/appi.neuropsych.12060141.
122. Kaur, D.; Ajinkya, S.; Nabi, J.; Manzoor, A.; Ghildiyal, R. Meningioma Masquerading as Acute Psychosis with Symptoms of Schizophrenia. *Bombay Hospital Journal* **2012**, *54*, 316–318.
123. Bhattacharya, S.K.; Satyan, K.S.; Ramanathan, M. Experimental Methods for Evaluation of Psychotropic Agents in Rodents: II-Antidepressants. *IJEB Vol.37(02) [February 1999]* **1999**.
124. Ciobanu, A.M.; Lisievici, M.G.; Coman, T.C.; Ciubotaru, G.V.; Drăghia, A.; Drăghia, F.; Ciucu, A.A. Giant Wing Sphenoid Meningioma with Principal Manifestation Depression. *Rom J Morphol Embryol* **2009**, *50*, 713–717.
125. Madhusoodanan, S.; Danan, D.; Brenner, R.; Bogunovic, O. Brain Tumor and Psychiatric Manifestations: A Case Report and Brief Review. *Ann Clin Psychiatry* **2004**, *16*, 111–113.
126. Lagares, A.; de Toledo, M.; González-León, P.; Rivas, J.J.; Lobato, R.D.; Ramos, A.; Cabello, A. [Bilateral thalamic gliomas: report of a case with cognitive impairment]. *Rev Neurol* **2004**, *38*, 244–246.
127. Chaskis, C.; Buisseret, T.; Michotte, A.; D'Haens, J. Meningioma of the Fourth Ventricle Presenting with Intermittent Behaviour Disorders: A Case Report and Review of the Literature. *J Clin Neurosci* **2001**, *8 Suppl 1*, 59–62, doi:10.1054/jocn.2001.0879.
128. Kellner, M.; Hirschmann, M.; Wiedemann, K. Panic Attacks Caused by Temporal Tumors: An Exemplary New Case and a Review. *Depress Anxiety* **1996**, *4*, 243–245, doi:10.1002/(SICI)1520-6394(1996)4:5<243::AID-DA7>3.0.CO;2-E.
129. Fulton, J.D.; Duncan, G.; Caird, F.I. Psychiatric Presentation of Intracranial Tumour in the Elderly. *International Journal of Geriatric Psychiatry* **1992**, *7*, 411–418, doi:10.1002/gps.930070606.
130. Ribeiro, S.A.; Oliveira-Souza, R.; Alvarenga, H. Bonnet Syndrome and Posterior Parasagittal Tumor: Clues to Neural Mechanisms. *Arq Neuropsiquiatr* **1989**, *47*, 230–234.
131. Goldstein, M.Z.; Richardson, C. Meningioma with Depression: ECT Risk or Benefit? *Psychosomatics* **1988**, *29*, 349–351, doi:10.1016/S0033-3182(88)72377-4.
132. Greenberg, L.B.; Mofson, R.; Fink, M. Prospective Electroconvulsive Therapy in a Delusional Depressed Patient with a Frontal Meningioma. A Case Report. *Br J Psychiatry* **1988**, *153*, 105–107.

133. Ghadirian, A.M.; Gauthier, S.; Bertrand, S. Anxiety Attacks in a Patient with a Right Temporal Lobe Meningioma. *J Clin Psychiatry* **1986**, *47*, 270–271.
